# Nitric oxide dependent metabolic and genetic dysregulation in metastatic triple-negative breast cancer

**DOI:** 10.1016/j.jbc.2026.113526

**Published:** 2026-09-08

**Authors:** Jennifer N. Daw, Pricila K. Moly, Su Chung, Sarah Young, Nhi Van, Sara Centuori, David G. Besselsen, Nathan Ellis, Ronald L. Heimark, William R. Montfort

**Affiliations:** 1 University of Arizona Cancer Center, Tucson, Arizona, USA; 2 CBIO Graduate Interdisciplinary Program, University of Arizona, Tucson, Arizona, USA; 3 Department of Chemistry and Biochemistry, University of Arizona, Tucson, Arizona, USA; 4 University Animal Care, University of Arizona, Tucson, Arizona, USA; 5 Department of Cellular and Molecular Medicine, University of Arizona, Tucson, Arizona, USA; 6 Department of Surgery, University of Arizona, Tucson, Arizona, USA

**Keywords:** inflammation, nitric oxide, metabolism, epigenetics, immunosuppression

## Abstract

Chronic inflammation drives many diseases, including cancer, where inflammation is associated with metastasis, the cause of death in 90% of cancer fatalities. Tumor inflammation drives inducible nitric oxide synthase (NOS2) and cyclooxygenase 2 (COX2) expression, each of which is associated with poor outcomes in cancer. Here, we knocked out the *NOS2* gene in the murine triple-negative breast cancer allograft tumor model 4T1 and examined metastatic spread from primary mammary tumors to lung in BALB/c mice with intact immune systems. Remarkably, while the parental 4T1 tumors were highly metastatic, metastasis from 4T1 *NOS2*^−/−^ tumors was nearly eliminated. In cell culture, we find that nitric oxide (NO) from NOS2 induces a glycolytic phenotype, epitrascriptomic dysregulation, and prostaglandin E_2_ synthesis by stimulating COX2. Cytokine addition led to DNA damage, presumably from NO. Human colorectal cancer cell line DLD1 yielded similar results. RNA sequencing of 4T1 cells revealed an NOS2-dependent reduction in mRNAs associated with regulation of chromosome and DNA replication, including cell cycle checkpoints, as well as mRNAs associated with RNA demethylation, including tRNA demethylation. Immunoprofiling of the tumor microenvironment revealed immunosuppressed primary tumors that were low in cytotoxic T cells and high in myeloid-derived suppressor cells expressing interferon gamma, which induces NOS2 expression. Taken together, these data suggest high NOS2 in tumor cells may drive metastasis through a broad accumulation of cancer-associated factors—including metabolic dysregulation, genetic dysregulation, and an increase in inflammatory prostaglandins—all of which contribute to aggressive cancers.

Chronic inflammation drives many diseases, including cancer, but the underlying mechanisms for disease enhancement are poorly understood (1). In cancer, inflammation is associated with metastasis, the spread of the disease from a primary tumor to secondary sites and ultimately the cause of death in 90% of cancer fatalities (2). One outcome of tumor inflammation is the induction of inducible nitric oxide synthase (iNOS or NOS2) and cyclooxygenase 2 (COX2), each of which is associated with poor outcomes in cancer and are the targets for clinical trials to treat cancer (3).

We seek to uncover underlying mechanism in NOS2/COX2 driven cancer. Multiple mechanisms have been proposed for the roles of NOS2 and COX2 in cancer progression, including epigenetic changes in gene expression (4, 5, 6), metabolic dysregulation leading to aggressive glycolytic states (7, 8), driving of epithelial to mesenchymal transition (EMT) with consequent tumor escape (9, 10), and immune suppression, allowing tumor cells to escape clearance by both innate and adaptive immune cells (11, 12, 13, 14). In the present study, we focus on a triple-negative breast cancer (TNBC) model called 4T1 to begin uncovering how NOS2 drives tumor metastasis. 4T1 cells were derived from a murine tumor allowing for study in immune-intact mice (15, 16). They display a TNBC phenotype, with loss of three typical breast cancer drug targets, the estrogen receptor, progesterone receptor, and human epidermal growth factor receptor 2. TNBC is aggressive and metastatic and has no targeted therapies (17); 4T1 allografts display these same characteristics.

Nitric oxide (NO) is produced by nitric oxide synthases 1-3 (NOS1-3) in mammals for signaling and regulation of a diverse set of physiological functions (18). NOS3 is constitutively expressed in the vasculature to regulate blood pressure, wound healing, sexual function and related activities. NOS1 is constitutively expressed in neuronal tissues to regulate nerve cell signaling and memory formation. Both NOS1 and NOS3 are regulated by calcium release into the cytosol and produce low (nanomolar) levels of NO. NO signal transduction at low levels is primarily through binding to soluble guanylyl cyclase (sGC), a ferrous heme protein that binds NO extremely tightly (picomolar affinity, (19, 20, 21)), leading to a large change in conformation, the stimulation of cyclic GMP activity, and the initiation of a signaling cascade with physiological outcomes. NO can also react with biomolecules to produce, for example, nitrotyrosine (22), nitrated lipids (23), and *S-*nitrosated/nitrosylated cysteine (24), all of which have additional signaling outcomes.

In contrast to NOS1 and NOS3, NOS2 is induced in immune cells in response to infection and inflammatory cytokines, reaching much higher levels of NO production (up to micromolar (25)). At these higher levels, NO is more reactive and toxic properties predominate. NO is a free radical with an unpaired electron that allows it to react with many organic biomolecules including protein, lipids, and DNA as well as with oxygen and metals such as iron. Elevated NO levels from NOS2 thus serves as one of several means for killing infectious pathogens by immune cells.

Curiously, NOS2 is induced in many non-immune cells in response to inflammatory cytokines, but to what benefit is unclear. Tumors are often under inflammatory stress due to intrinsic factors such as high proliferation rates and increasing mutation burden, as well as extrinsic factors involving the tumor microenvironment, necrosis, hypoxia and related factors, which promote the recruitment of immune cells and release of inflammatory cytokines (26). We have recently shown that NOS2 induction and NO production in murine TNBC tumor cells 4T1 and human colorectal cancer cells DLD1 is surprisingly high, reaching the same high levels as macrophages, which are part of the innate immune system for fighting infection (27). Others have shown that high levels of tumor NOS2 correlate with more aggressive tumors and poor outcomes (3). NO from both immune cells and tumor cells appear to contribute to disease progression (28). NO-dependent tumor enhancement does not appear to involve signaling through sGC since sGC expression is suppressed in tumor cells (29) and cGMP signaling is, in fact, thought to be tumor suppressing (30). It seems likely, therefore, that the reactive properties of NO at higher concentration drives tumor aggression.

Here, we pursue underlying mechanism in NOS2 promotion of disease progression when expressed in tumor cells. We generated a *NOS2* gene knockout in cell line 4T1 and characterized its cellular properties and propensity to metastasize. Remarkably, *NOS2*^−/−^ 4T1 cells formed primary tumors that were nearly metastasis free. We examined NOS2-dependent changes in tumor cell metabolism, epigenetics, gene expression, COX2 enhancement, and the tumor microenvironment to begin understanding how NOS2-drives metastasis. Advantages to our approach include (a) the use of naturally occurring NO rather than external NO delivery or NOS2 overexpression; (b) knockout of the *NOS2* gene to focus on NOS2 specific cellular changes; and (c) the ability to examine metastasis in immune competent mice. Our data indicate that NOS2-derived NO alters metabolism, gene expression, prostaglandin production, and DNA damage response that, together, promote the aggressive nature of NOS2 expressing tumors.

## Results

### IFNγ drives NOS2 expression in tumor cells through the JAK/STAT pathway

We previously showed that NOS2 expression in tumor cell lines 4T1 and DLD1 is negligible in the absence of cytokine stimulation, but upon stimulation, NOS2 levels and NO production reach levels as high as that for the murine macrophage cell line RAW 264.7, reaching NO production values > 1 pmol NO/min/μg total cellular protein (27). The *NOS2* promotor has response elements for transcription factors STAT, NFκB, and HIF1α (31). In 4T1 cells, interferon gamma (IFNγ), an activator of the JAK/STAT pathway, provided the largest stimulus for NOS2 expression, with interleukin 1β (IL1β) and tumor necrosis factor α (TNFα), activators of the NFκB signaling pathway, each providing an additional boost. We previously showed that hypoxia, which activates transcription factor HIF1α, has only a small effect on NOS2 induction in 4T1 cells (27). Here, we show that NOS2 induction by IFNγ and TNFα yield maximal NOS2 expression after ∼8 h (Fig. 1*A*) and that both STAT1 and STAT3 are activated through phosphorylation, with phosphorylated STAT3 (pSTAT3) levels increasing first, peaking at ∼4 h, and pSTAT1 levels peaking at ∼8 h. NOS2 induction is completely abrogated with the JAK inhibitor ruxolitinib for both mRNA (Fig. 1*B*) and protein (Fig. 1, *C* and *D*), consistent with the primary role of IFNγ in NOS2 expression.

**Figure 1:**
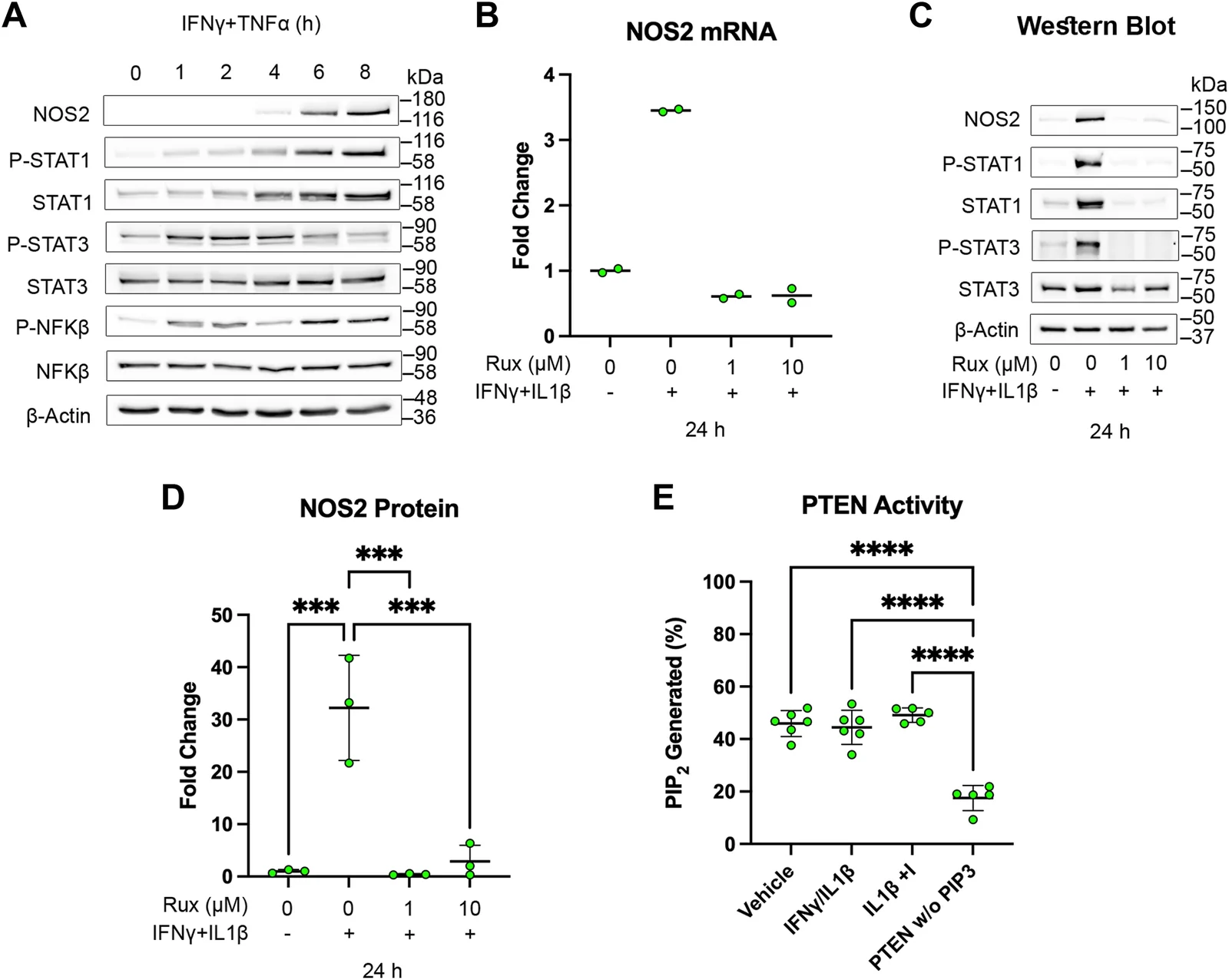
**Signaling pathways affecting NOS2 expression in 4T1 cells.***A*, Western blots for proteins signaling through IFNγ and IL1β. Molecular weight markers above and below the band of interest are shown. Full gels are shown in Figure S3. (*B*) *NOS2* mRNA relative to β-actin measured by qPCR (2ˆΔΔCT). Ruxolitinib (Rux), a Janus kinase (JAK) inhibitor, eliminated *NOS2* mRNA induction. *C*, Western blots for STAT and NOS2 proteins. STAT1, phosphorylated STAT1 (pSTAT1), pSTAT3, and NOS2 increased in response cytokines. Inhibition of JAK by ruxolitinib overcomes these increases, indicating NOS2 increases are primarily due to IFNγ and the JAK/STAT signaling pathway. *D*, Quantification of NOS2 levels by Western blot (n = 3 independent replicates). Statistical significance was estimated by ordinary one-way ANOVA with Tukey’s multiple comparison’s test. Indicated *p* values (*left* to *right*) are 0.0004, 0.0003, and 0.0006. *E*, phosphatase and tensin homolog (PTEN) activity in the presence or absence of NOS2 stimulation (n = 6 independent biological replicates; independent biological replicates are the average of two technical replicates), measured by ELISA upon addition of substrate phosphatidylinositol (3,4,5)-triphosphate (PIP_3_). Statistical significance was estimated by ordinary one-way ANOVA with Tukey’s multiple comparison’s test; where indicated, *p* value < 0.0001. Activity was unaffected by cytokine stimulation or NOS2 inhibitor 1400W (+I).

One possibility for how excess NO leads to a change in tumor cell aggression is the activation of the Akt signaling pathway, which is increased in many cancers (32) and has been implicated in breast cancer (33). This pathway is initiated through stimulation of phosphatidylinositol 3-kinase (PI3K), yielding phosphatidylinositol triphosphate (PIP_3_) and leading to phosphorylated Akt (pAkt) and downstream signaling for tumor growth and proliferation. This activity is opposed by phosphatase PTEN, a tumor suppressor that returns PIP_3_ to PIP_2_ and can be inhibited by externally delivered *S*-nitrosocysteine *via S-*nitrosation of Cys 83 in HEK293T cells (34). To evaluate NOS2-dependent PTEN inhibition in 4T1 cells, we examined PTEN activity after immunoprecipitation from 4T1 cell lysates using a colorimetric competitive ELISA assay measuring PIP_3_ conversion to PIP_2_. Protein was isolated and PIP_2_ generation undertaken in the absence of dithiothreitol (DTT) to preserve any *S*-nitrosated cysteines on PTEN. Similar levels for PTEN activity were seen with untreated cells, cells treated with cytokines, and cells treated with a NOS2 inhibitor (Fig. 1*E*). It therefore appears PTEN is not inhibited by endogenously produced NO in 4T1 cells, unlike when *S*-nitrosocysteine is exogenously added to HEK293T cells (34). Additional studies are needed to evaluate whether PTEN is in general unaffected by endogenously produced NO across cell types.

### NOS2^−/−^ 4T1 tumor cells lack metastasis

To isolate the contribution of NOS2 to tumor progression, we used CRISPR/Cas9 to knock out the *NOS2* gene, yielding two independent clones of homozygous 4T1 NOS^−/−^ cell lines, referred to here as KO1 and KO2. We examined *NOS2* transcript, protein and NO levels in response to cytokines in each cell line and found only the parental 4T1 cells produced NOS2 and NO, as expected (Fig. S1). Additionally, parental NO production was completely abolished with NOS2-specific inhibitor 1400W (Fig. S1*C*), as previously demonstrated (27, 35).

Since 4T1 cells are syngeneic with BALB/c mice, we were able to examine tumor growth in mice having intact immune systems. Allograft tumors were formed by injection of 4T1 parental or knockout cells into the mammary fat pads of BALB/c mice and allowed to grow for 3 to 4 weeks, followed by sacrificing of the mice and examining the primary mammary tumor and lung metastases. Primary tumors for all three cell lines grew similarly, varying widely in size but reaching similar average sizes (Fig. 2*A*). Tumor metastasis was evaluated from representative hematoxylin and eosin (H&E) stained lung tissue sections (Fig. 2, *B* and *C*), 1 to 2 sections per lung with metastasis count averaged where two sections were examined. Parental 4T1 primary tumors extensively metastasized to the lung, as expected. However, remarkably, lung tissue from mice with 4T1 *NOS2*^−/−^ tumors were nearly metastasis free.

**Figure 2:**
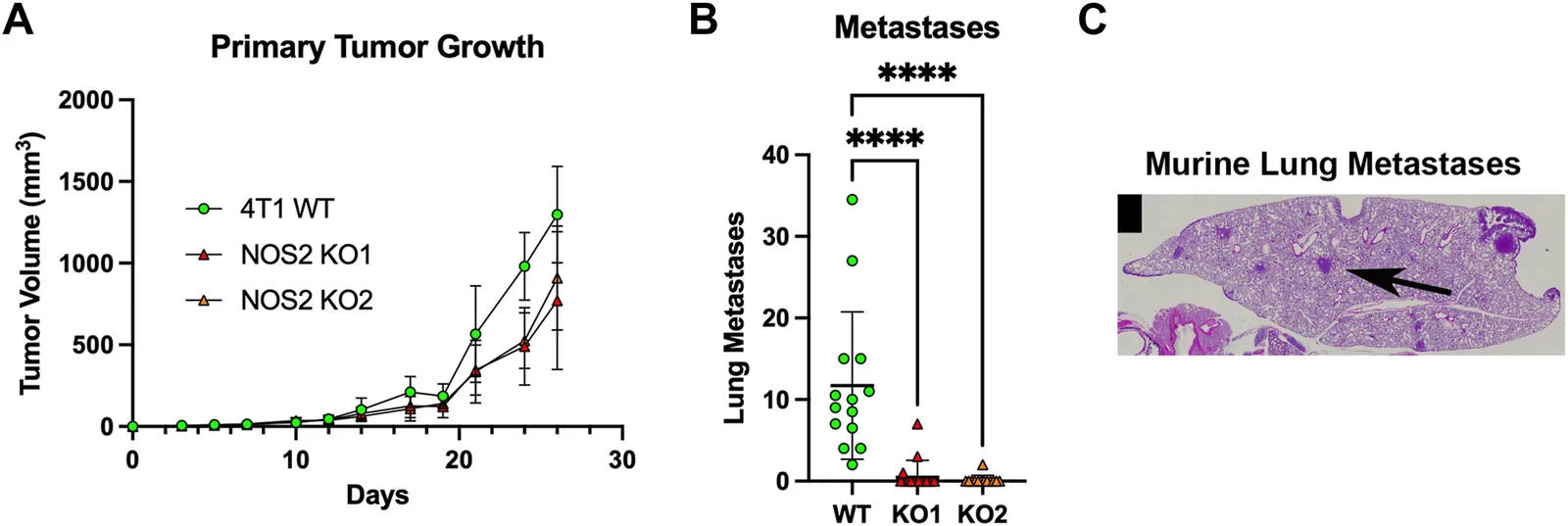
**Tumor growth and metastasis for 4T1 wild type and *NOS2* knockout allografts in mice.***A*, Representative primary mammary tumor growth for wild type (WT) and *NOS2* knockout cell lines KO1 and KO2. Average and standard deviation are shown for eight mice per group. No statistical difference in tumor growth was detected. *B*, lung metastases quantified from 1 to 2 representative hematoxylin and eosin (H&E) stained lung sections, n = 13 to 17 mice per group from two independent experiments. Where two sections were evaluated, metastasis count was averaged. WT tumors were highly metastatic while KO tumors were nearly without metastases. Statistical evaluation was by ordinary one-way ANOVA with Tukey’s multiple comparison test; where indicated, *p* value < 0.0001. *C*, representative H&E-stained lung section exhibiting metastatic lesions in a mouse with a WT 4T1 primary tumor. The arrow indicates a typical metastatic tumor of several seen in the image.

In primary tumors, NOS2 expression may derive not only from tumor cells but also from other cell types in the tumor microenvironment such as tumor associated immune cells and endothelial cells from tumor vasculature. We measured NOS2 expression in tumor lysates by Western blot and found NOS2 expression in both parental 4T1 and in 4T1 *NOS2*^−/−^ tumors (Fig. S2). Overall NOS2 levels were quite variable in these bulk tumor measurements, reflecting the complicated tumor microenvironments. We also examined tumor angiogenesis by counting the average number of capillary cross sections per 200× field, from which we concluded angiogenesis was mild overall and did not vary greatly between parental and NOS2 KO tumors. Likewise, tumor necrosis varied wildly across samples, without any trend based on NOS2 tumor cell status.

### 4T1 primary tumors are immunosuppressed

The tumor microenvironment plays a critical role in tumor expansion and escape. Tumor cells are generally cleared by the immune system unless blocked or inhibited. In many cancers, tumor cells produce factors that induce immune suppression, leading to immune evasion and cancer progression (36). Several promising new drugs, including the PD-L/PD-L1 and CTLA-4 immune checkpoint inhibitors, function by blocking immune cell suppression, allowing for the natural clearance of tumors (37). In breast cancer, however, these drugs have seen only mixed success (38).

To determine if NOS2 plays a role in damping the immune response, we examined the immune cell status in parental and *NOS2* KO 4T1 primary tumors grown in immunocompetent BALB/c mice. Immune cells were isolated from freshly dissociated 4T1 tumors, stained for 18 immune cell markers, and examined by flow cytometry. We found both parental and *NOS2*^−/−^ tumors to be immunosuppressed (Table 1). Myeloid cells and T cells were roughly equal in number but cytotoxic T cells and activated helper T cells were low in number. Activated regulatory T cells, which inhibit T cell activation, were present. Immunosuppressive myeloid-derived suppressor cells (MDSC) were abundant with monocytic (Mo-MDSCs) and polymorphonuclear (PMN-MDSCs) roughly equal in number. Immunosuppressive M2 macrophages (arginase-1+) predominated over antitumor M1 macrophages (NOS2+). Multiple immune cells expressed IFNγ, most notably MDSCs, presumably leading to NOS2 expression in the tumor. This tumor immune cell profile is consistent with a suppressed TME (36) and similar to another recent report on the 4T1 tumor immune environment (39).

**Table 1:** Immune cell populations from flow cytometry

Leukocyte and myeloid cells				
Cell Type^a^	Leukocyte	Myeloid	Mo-MDSC	PMN-MDSC
Gating Markers^b^	CD45+, (Cell Count)^c^	CD45+, CD11b+	CD45+, CD11b+, Ly6C+, Ly6G-	CD45+, CD11b+, Ly6C (low/-), Ly6G+
WT^d^	25,215	64 (6)	11 (1)	6 (5)
KO1	40,549	50 (4)	13 (0)	9 (5)
KO2	20,427	67 (14)	8 (4)	13 (2)

T Cells					
Cell Type^a^	T Cells	Cytotoxic T Cells	Helper T Cell	Helper T Cell, Activated	Regulatory T Cell, Activated
Gating Markers^b^	CD45+, CD3+	CD45+, CD3+, CD8+, CD4-	CD45+, CD3+, CD4+, CD8-	CD45+, CD3+, CD4+, CD8-, CD25+	CD45+, CD3+, CD4+, CD8-, CD25+, FoxP3+
WT^d^	46 (9)	13 (<1)	9 (2)	4 (1)	3 (<1)
KO1	64 (5)	24 (12)	10 (3)	5 (2)	3 (<1)
KO2	62 (7)	21 (13)	11 (1)	5 (1)	5 (1)

Macrophage, Dendritic, Neutrophil, and Natural Killer Cells				
Cell Type^a^	Macrophage	Dendritic	Neutrophil	Natural Killer
Gating Markers^b^	CD45+, F4/80	CD45+, CD11c+	CD45+, CD11b+, CD3-, Ly6C+, Ly6G+	CD45+, CD3-, perforin+
WT^c^	27 (10)	3 (1)	2 (1)	7 (<1)
KO1	7 (1)	3 (<1)	2 (1)	10 (1)
KO2	13 (3)	1 (<1)	7 (2)	2 (<1)

Cells Expressing IFNγ					
Cell Type^a^	IFNγ in Leukocytes	IFNγ in Mo-MDSC	IFNγ in PMN-MDSC	IFNγ in Neutrophils	IFNγ in Macrophage
Gating Markers^b^	CD45+, IFNγ+	CD45+, CD11b+, Ly6C+, IFNγ+	CD45+, CD11b+, Ly6G+, IFNγ+	CD45+, CD11b+, CD3-, Ly6C+, Ly6G+, IFNγ+	CD45+, CD3-, CD11b+, F4/80+, IFNγ+
WT^d^	9 (1)	6 (4)	5 (4)	<1 (<1)	8 (2)
KO1	8 (1)	5 (1)	3 (2)	<1 (<1)	3 (1)
KO2	14 (2)	12 (1)	13 (2)	1 (<1)	2 (1)

Cells Expressing NOS2			
Cell Type^a^	Mo-MDSC	Neutrophils	Macrophage
Gating Markers^b^	Mo-MDSC and NOS2+	Neutrophil and NOS2+	Macrophage and NOS2+
WT^d^	<1 (<1)	<1 (<1)	2 (1)
KO1	<1 (<1)	<1 (<1)	1 (1)
KO2	<1 (<1)	1 (<1)	<1 (<1)

Cells Expressing ARG1				
Cell Type^a^	Mo-MDSC	PMN-MDSC	Neutrophil	Macrophage
Gating Markers^b^	Mo-MDSC and ARG1+	PMN-MDSC and ARG1+	Neutrophil and ARG1+	Macrophage and ARG1+
WT^d^	<1 (<1)	<1 (<1)	<1 (<1)	9 (9)
KO1	4 (1)	1 (<1)	1 (<1)	4 (<1)
KO2	3 (1)	1 (<1)	1 (<1)	10 (3)

a Abreviations: MDSC: myeloid-derived suppressor cell; Mo: monocytic; PMN: polymorphonuclear; TAM: tumor associated macrophage; ARG1: arginase 1.

b Gating strategy: cells included are gated for live single cells followed by the listed immune cell markers.

c Average cell count for two independent measurements.

d Percentage of total leukocytes, average and (standard deviation), for two independent measurements.

Although the immune profiles for parental and KO tumors were similar, there were trends in the data suggesting *NOS2* knockout may lead to reduced immune suppression. For example, cytotoxic T cells increased in KO1 and KO2 while macrophages decreased; however, additional analyses will be required to determine the statistical significance of these differences. Nonetheless, although there may be a helpful trend with loss of NOS2, it seems likely that the increased metastasis of the parental cells is due to some other factor. Additionally, our analysis is for bulk tumor and larger changes in immune response due to loss of NOS2 might be present in a subset of cells, for example, at the tumor margin (3).

### Oxidative phosphorylation is suppressed in NOS2-expressing cells

Tumors are known to have increased reliance on glycolysis for energy despite having oxygen available for oxidative phosphorylation, referred to as aerobic fermentation and initially observed by Otto Warburg and colleagues (40). Nitric oxide has been shown to induce a glycolytic phenotype in macrophages through interfering with pyruvate dehydrogenase and aconitase in the tricarboxylic acid (TCA) cycle, which feeds NADH and FADH into oxidative phosphorylation (28, 41). NO can also directly inhibit complex IV of oxidative phosphorylation through binding to its heme moiety (42, 43) and indirectly through reacting with O_2_, generating a hypoxic environment. Other sites in the electron transport chain are sensitive to NO-dependent peroxynitrite (44).

To further examine NO-dependent changes in tumor cells, we measured metabolic production using a Seahorse instrument. We examined murine 4T1 parental and *NOS2* KO cells in addition to human DLD1 cells in the presence or absence of cytokine addition, and in the presence or absence of NOS2 inhibitor 1400W, determining ATP production from oxidative phosphorylation and from glycolysis, which account for essentially all ATP produced. We present these data in Figure 3 as total ATP and percentage of ATP from glycolysis. For parental 4T1 cells, induction of NOS2 with cytokines leads to an 18% increase in ATP from glycolysis, reaching, on average, 66% of all ATP produced (Fig. 3*A*), with oxidative phosphorylation contributing only 34%. Addition of NOS2 inhibitor 1400W shifted the glycolytic percentage back down below 50%. *NOS2* KO cells showed no change in glycolytic percentage in the presence of cytokine. Taken together, these data indicate NO from NOS2 directly inhibits oxidative phosphorylation. Human colorectal cancer cell line DLD1 displayed a similar change but with an even greater increase in ATP coming from glycolysis (27%, Fig. 3*C*), yielding a total of 73% of ATP coming from glycolysis.

**Figure 3:**
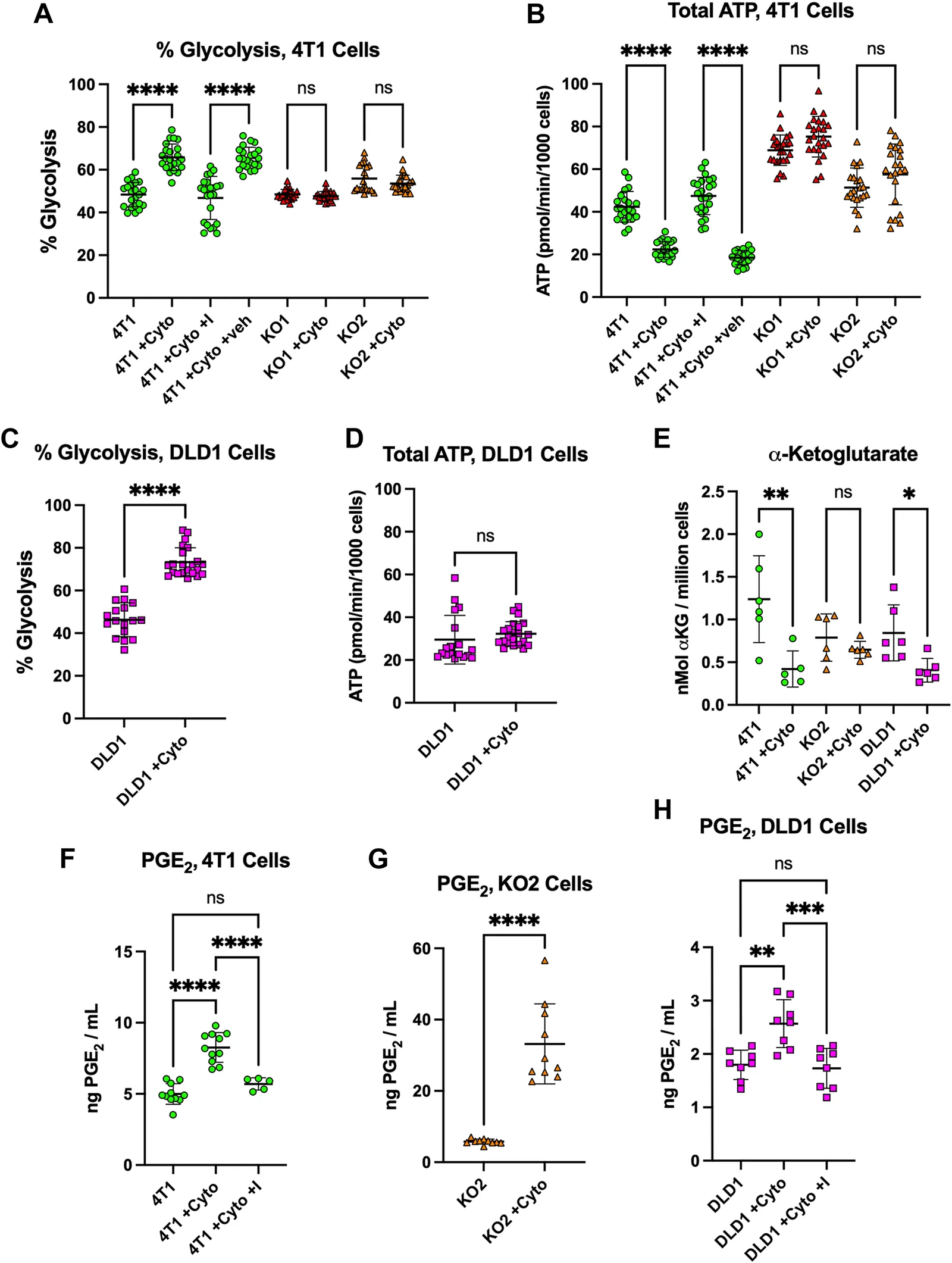
**Metabolic changes in 4T1 and DLD1 cells upon NOS2 induction.***A*, ATP production from glycolysis and oxidative phosphorylation was measured in real time using a Seahorse analyzer. Shown is the percentage of total ATP production generated by glycolysis in 4T1 WT and KO cells in the presence or absence of cytokine addition (+Cyto, IFNγ and IL1β) and NOS2 inhibitor 1400W (+I). Each condition was independently measured 6 to 16 times on two different days, yielding n = 21 to 24 independent measurements per condition. NOS2 activity inhibited oxidative phosphorylation, increasing the glycolytic percentage by ∼17% (66% overall). NOS2 inhibition returned the glycolytic percentage to ∼48%. No change in glycolytic percentage was seen in *NOS2* KO cells. Statistical significance was estimated by ordinary one-way ANOVA. *B*, total ATP production from the cells shown in (*A*). Total ATP production dropped by ∼50% in 4T1 WT cells in the presence of active NOS2, but not in *NOS2* KO cells. *C*, Glycolysis in DLD1 cells increased by ∼20% on NOS2 induction by cytokines IFNγ, IL1β, and TNFα (n = 17–22 independent measurements). (*D*) Total ATP production from the cells shown in (*C*). Total ATP was unchanged by NOS2 induction in DLD1 cells. *E*, Levels for α-ketoglutarate were measured by mass spectrometry and found to be lowered by >50% upon NOS2 induction in 4T1 and DLD1 cells, but not in *NOS2* KO cells (n = 6 independent measurements). Statistical significance was estimated by Welch’s *t* test. Indicated *p* values are 0.021, 0.281 (ns), and 0.0092. (*F*–*H*) PGE_2_ levels were measured by competitive ELISA for 4T1 WT, *NOS2* KO2, and DLD1 cells (n = 5–11 biological replicates and include two independent experiments; biological replicates are the average of two technical replicates). Statistical significance was estimated by ordinary one-way ANOVA with Tukey’s multiple comparison’s test in *panel H* with *p* values (*left* to *right*) of 0.0013, 0.93 (ns), and 0.0006. 4T1 and DLD1 cells exhibited an ∼50% increase in PGE_2_ levels upon cytokine addition, which was reversed on addition of NOS2 inhibitor 1400W. *NOS2* KO cells displayed an ∼6-fold increase PGE_2_ by an unknown mechanism. Statistical significance was estimated by ordinary one-way ANOVA with Tukey’s multiple comparison’s test (*panels A, B and F*) or Welch’s *t* test (*panels C and G*), with indicated *p* values < 0.0001.

For total ATP, the two cell lines differed. 4T1 cells displayed higher ATP production than DLD1 cells, consistent with the very fast 4T1 growth rate. NOS2 lowered total ATP in 4T1 cells (Fig. 3*B*), with both oxidative phosphorylation and glycolysis inhibited overall, but with glycolysis nonetheless providing an overall greater percentage of ATP produced. In contrast, DLD1 cells retained the same, somewhat lower level of ATP production regardless of NOS2 status (Fig. 3*D*).

RAW 264.7 murine macrophage cells display lower levels of metabolite α-ketoglutarate when NOS2 is expressed, which prior studies attributed to inhibition of aconitase and pyruvate dehydrogenase (28, 41). To determine whether this also occurs in tumor cells, we measured α-ketoglutarate levels in 4T1 and DLD1 cells using mass spectrometry (Fig. 3*E*). Cytokine stimulation reduced α-ketoglutarate levels as much as 3-fold in parental 4T1 and DLD1 cells compared to unstimulated cells. Cytokine addition to *NOS2* KO cells had no effect on α-ketoglutarate levels (Fig. 3*E*) while addition of inhibitor 1400W to 4T1 parental and DLD1 cells partially restored the α-ketoglutarate levels (not shown). Loss of α-ketoglutarate not only suppresses oxidative phosphorylation but, critically, also removes a key substrate for epigenetic demethylation, contributing to epigenetic dysregulation (described below).

### NOS2 stimulates cyclooxygenase 2 and increases inflammatory prostaglandins

Cyclooxygenase 2 (COX2), also called prostaglandin endoperoxide synthase (PGHS2, gene name *PTGS2*), is another inflammatory enzyme induced by IFNγ and other cytokines (45) that is also associated with poor outcomes in breast cancer (3). Intriguingly, NO can stimulate COX2 activity (46). Although the mechanism of activation is incompletely understood, it may be through reaction with peroxynitrite, the product of nitric oxide and superoxide, which can act as a peroxide substrate for COX2 (46), activating the enzyme from its resting state by generating the tyrosyl radical needed for catalysis (47).

To determine the role of NOS2 in stimulating tumor cell COX2, we examined NOS2-dependent COX2 activity in 4T1 parental, *NOS2* KO (KO2), and DLD1 cells, using a commercial competitive ELISA kit for measuring PGE_2_ production. PGE_2_ levels were high under all conditions (Fig. 3*F*), suggesting COX2 in cancer cells is constitutively expressed (confirmed by RNA-seq, Table 2, described below). Parental 4T1 (Fig. 3*F*) and *NOS2* KO (Fig. 3*G*) cells released similar PGE_2_ levels in the absence of cytokine, ∼5 ng/ml of cell culture, while DLD1 (Fig. 3*H*) cells released ∼2 ng/ml. Addition of cytokine increased PGE_2_ levels by ∼50% in both parental 4T1 and DLD1 cells, an increase that appears to be directly due to NO production since addition of NOS2 inhibitor 1400W overturned the increase. These data suggest NOS2 and COX2 are interdependent drivers of cancer.

**Table 2:** Selected message levels from RNA-seq

Cell	CPM *NOS2*^a^	Fold Change^b^	P value^c^	CPM *COX2*	Fold change	*p* Value	CPM *H2AX*	Fold change	*p* value
KO1	NA	–		651	–		111	–	
KO1 +cyto	NA	–		685	1.0	ns	75	1.5	0.03
4T1	132	–		954	–		107	–	
4T1 +cyto	1084	−8.2	0.0002	686	1.4	ns	14	7.6	<0.0001
4T1 +cyto, +I^d^	805	−6.1	0.006	714	1.3	ns	48	2.2	0.0003
DLD1	1.5	–		5.2	–		124	–	
DLD1 +cyto	223	−149	0.0003	2.8	1.9	ns	94	1.3	ns
DLD1 +cyto, +I	189	−126	0.01	2.9	1.8	ns	94	1.3	ns

a Average counts per million reads (n = 3).

b Larger value/smaller value with “-“ indicating a larger value in the denominator.

c *p* value on comparing to untreated cell of the same type, from one-way ANOVA with Tukey’s multiple comparison test or *t* test. ns: not significant.

d I: NOS2 inhibitor 1400W.

Surprisingly, cytokine addition to *NOS2* KO2 cells led to a much larger (∼6-fold) increase in PGE_2_, despite the lack of NO production in these cells (Fig. 3*G*). The reason for this increase is unclear. Although the COX2 gene displays epigenetic regulation that might be sensitive to NO production (48), *NOS2* KO1 cells did not display an increase in COX2 message upon cytokine addition as assessed by RNA-seq (described below; Table 2), suggesting the increase in COX2 activity in KO2 cells is due to some other change in regulation, such as reduced COX2 protein degradation or some other factor independent of NOS2 status.

### NOS2-expressing cells undergo epigenetic changes

Gene expression is influenced by changes in epigenetic markers, including DNA, RNA, and histone methylation, and histone acetylation (49). In cancer, changes in epigenetic markers drive gene expression changes and aggressive cancer phenotypes (50, 51, 52). We and others have previously shown that excess NO inhibits RNA and DNA demethylases, giving rise to changes in oncogenic gene expression (5, 6). Despite these promising studies, how much NO is required remains unknown, as is which altered genes give rise to metastasis (if any), and which demethylases are most sensitive to NO.

We examined 4T1 and DLD1 cells for NO-dependent changes in m^6^A status by ELISA. When exposed to cytokine, parental 4T1 and DLD1 cells displayed increased m^6^A levels, increasing by > 2-fold (Fig. 4*A*), while 4T1 *NOS2*^*−/−*^ cells did not. Upon inhibiting NOS2 with 1400W, parental 4T1 cells and DLD1 cells stimulated with cytokine no longer displayed increased m^6^A levels. Thus, physiological NO levels from endogenous NOS2 expression leads to epigenetic dysregulation in tumor cells.

**Figure 4:**
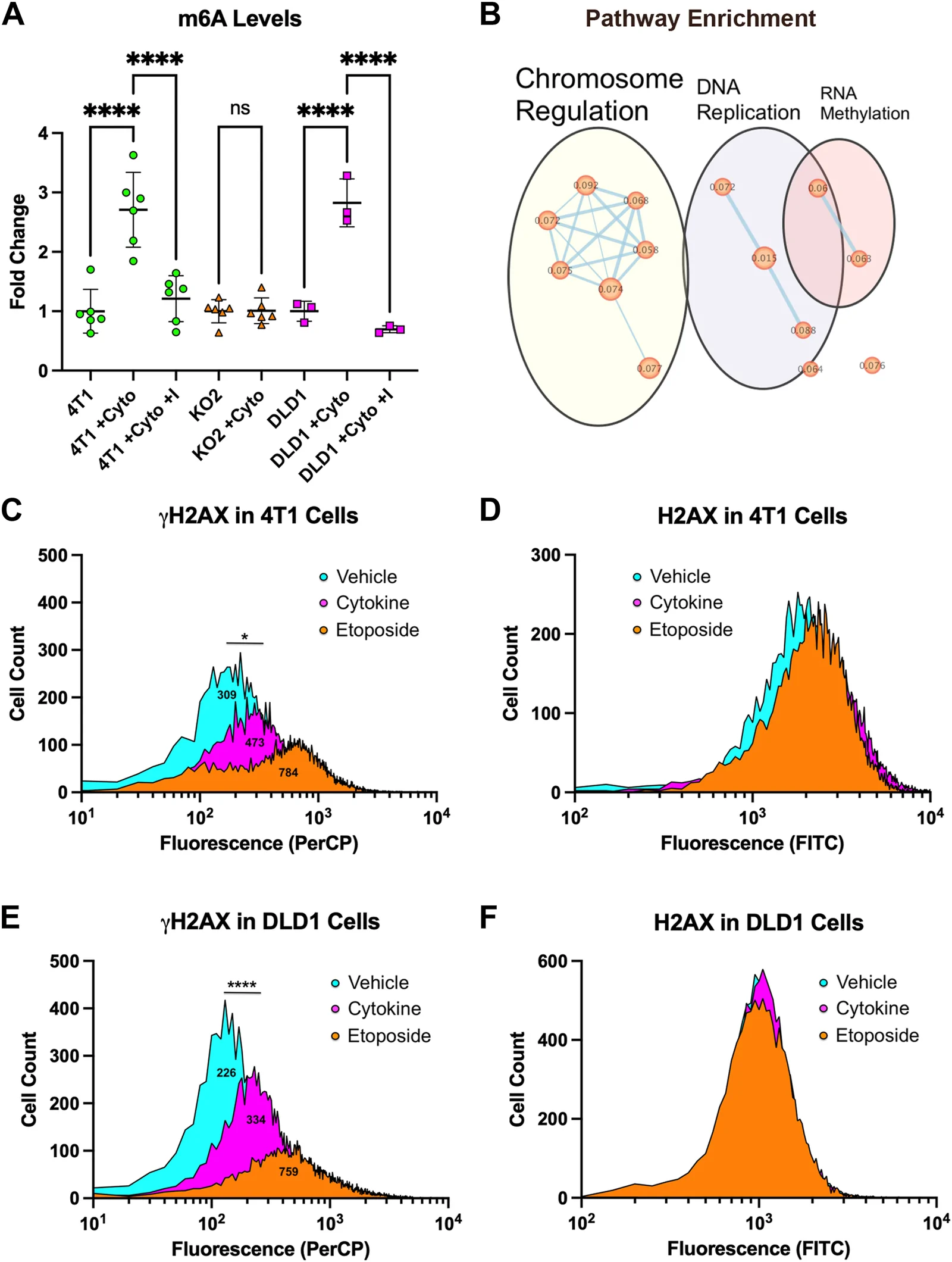
**Epigenetic and gene expression changes upon NOS2 induction.***A*, Changes in N^6^-methyladenosine (m^6^A) were measured by competitive ELISA and found to increase by nearly 3-fold in 4T1 WT and DLD1 cells after exposure to cytokine (+Cyto). These increases were reversed by addition of NOS2 inhibitor 1400W (+I). No increase was seen for *NOS2* KO2 cells (n = 6 independent measurements). (*B*) Gene set enrichment analysis for 4T1 WT and *NOS2* KO1 cells in the presence of cytokines IFNγ and IL1β. 14 gene sets forming three clusters were enriched in KO1 cells (q-value < 0.1). The q-value for each gene set node is shown on the node. Genes of importance for chromosome maintenance, cell cycle, and DNA replication were found in clusters 1 and 2; cluster three contained genes involved in RNA methylation. *C-F*, levels of total H2AX and phosphorylated H2AX (γH2AX) were measured by flow cytometry, using two-color detection. Shown are histograms of cell fluorescence populations. Cyokine addition led to an increase in γH2AX in both 4T1 and DLD1 cells. Addition of the DNA damaging agent etoposide induced greater damage, as expected. The median fluorescence is noted on each distribution. Statistical significance was estimated by one-way ANOVA assuming a nonparametric distribution (Kruskal-Wallis test), with *p* value = 0.0395 for 4T1 cells (*C*) and *p* value < 0.0001 for DLD1 cells (*E*). Total H2AX did not change across conditions.

### Transcriptome analyses implicate NO-dependent genomic instability

We undertook mRNA sequencing (mRNA-seq) to examine whether NO induced a distinct gene-expression profile linked to metastasis. For this, we examined WT 4T1, 4T1 *NOS2* KO1, and DLD1 cells in the presence and absence of cytokine and NOS2 inhibitor, eight conditions in all (Table S5). Pairwise differential gene expression was calculated using program edgeR (53). Pathway enrichment was evaluated with software packages Gene Set Enrichment Analysis (GSEA) (54, 55) and Cytoscape (56), focusing on the gene ontology biological process gene sets (57, 58). Addition of cytokine induced a large change in gene expression, as expected, and enriched pathways associated with cytokine-induced inflammation for all cell types. In contrast, gene expression profiles for *NOS2* KO1 and WT 4T1 cells in the absence of cytokine were very similar, displaying only modest changes in gene expression with no enriched gene sets identified.

Comparing *NOS2* KO1 and WT 4T1 cells after cytokine addition offered the possibility of identifying mRNA changes due to endogenous NO alone, controlling for changes due to cytokine addition. 14 enriched gene sets and three gene set clusters were identified with q-value < 0.1 in KO1 + cytokine when compared to WT 4T1 + cytokine (Fig. 4*B*). Ten of the enriched pathways concern DNA replication and cell cycle regulation (Tables S6 and S7), suggesting a possible loss of replication regulation in WT 4T1 cells and genomic instability, a hallmark of metastasis (59). Notable also were two enriched pathways indicating loss of RNA and tRNA methylation genes in WT 4T1 cells, consistent with the change in m^6^A levels described previously. On comparing WT 4T1 cells with and without cytokine addition, 25 enriched gene sets associated with DNA replication fidelity were identified in cells without cytokine, including the set of 10 associated with KO1 cells, again pointing to NO-induced genomic instability.

The other conditions tested did not provide clear insight from GSEA. On comparing WT 4T1 + cytokine with WT 4T1 + cytokine and NOS2 inhibitor, DNA replication fidelity gene sets were unchanged, possibly due to incomplete inhibition of NOS2 over the experimental time period (∼16 h) or to off-target effects of compound 1400W. Likewise, on comparing DLD1 cells with and without cytokine, loss of DNA replication fidelity gene sets was not seen, although three DNA damage response gene sets were enriched in the cytokine stimulated DLD1 cells. As with 4T1 cells, no significant change was observed on addition of inhibitor. Additional studies will be required to uncover which changes in mRNA levels are most clearly linked to metastasis.

Expression levels for *NOS2* and *COX2* were extracted from the RNA-seq data (Table 2). *NOS2* message levels in the RNA-seq analyses increase substantially in all cells when treated with cytokine, consistent with Western blot, NO measurement, and qPCR measurements (Figs. 1 and S1). In contrast, *COX2* message levels were high in 4T1 and 4T1 *NOS2* KO1 cells both in the absence and presence of cytokine, consistent with PGE_2_ measurements (Fig. 3, *F* and *G*) but unlike that previously reported for other breast cancer cells (45). Likewise, *COX2* was present in DLD1 cells under all conditions. *COX1* levels were low in 4T1 cells under all conditions, averaging 16 ± 13 CPM, and not found in DLD1 cells.

### Cytokine-induced DNA damage uncovered using flow cytometry

H2AX is a variant of histone H2A that is essential for recognizing DNA damage and assisting in assembling DNA damage repair protein complexes. In DNA repair, H2AX is phosphorylated to generate γH2AX, which provides a scaffold for assembly of DNA repair machinery (60, 61, 62). H2AX message was present under all conditions in our RNA-seq data, as expected, but was significantly reduced in 4T1 cells upon addition of cytokine, an effect partially reversed by addition of a NOS2 inhibitor (Table 2). In contrast, 4T1 *NOS2* KO1 cells displayed only a slight decrease in H2AX message levels upon cytokine addition, and DLD1 cells did not display any change in H2AX message levels upon addition of cytokine.

Nitric oxide has long been known to induce DNA damage (63). To examine whether DNA damage occurs in response to cytokine addition, we undertook a two-color flow cytometry experiment to simultaneously measure both H2AX and γH2AX, which indicated significant cytokine-dependent DNA damage in both 4T1 and DLD1 cells (Fig. 4, *C* and *D*), presumably in response to NO production. These data underly the importance of changes in chromosome regulation and DNA replication pathways found by mRNA-seq. Interestingly, H2AX protein levels did not change despite a strong signal for message change in the RNA-seq data.

## Discussion

Inflammation is a rising cause of disease progression and receiving considerable attention as a drug target, particularly in cancer, diabetes, cardiovascular disease, and aging. Yet the mechanisms underlying inflammation-driven disease progression are likely multifactorial and poorly understood. Here, we developed a murine TNBC model for examining the role of inflammation driven NO production in TNBC metastasis. Remarkably, we found that *NOS2* knockout severely inhibits metastasis in the 4T1 TNBC tumor model. The gross morphology of lungs from mice with tumors from WT 4T1 cells displayed extensive metastasis while those with NOS2 knocked out were nearly metastasis free. To quantify this difference, we counted metastatic lesions from representative lung sections (Fig. 2). While this count underestimates the total number of metastases to the lung, it clearly demonstrates the loss of metastasis when the NOS2 gene is absent.

To begin understanding the link between NOS2 activity and metastatic progression, we examined multiple factors that might be affected, focusing on endogenous NO production at natural levels rather than on exogenously delivered NO or NO from NOS2 overexpression. We find that the primary cytokine of importance for inducing NOS2 in 4T1 cells is IFNγ and that JAK inhibitor ruxolitinib completely blocks cytokine-dependent NOS2 production (Fig. 1). NOS2 levels are maximal 8 h after stimulation, displaying ∼3.5-fold increase in NOS2 message and ∼30-fold increase in NOS2 protein. PTEN, which dampens the AKT1 signaling pathway associated with some cancer progressions, and has been linked to NO-dependent inhibition, shows no sign of inhibition in 4T1 cells from NOS2 induction.

Interestingly, *NOS2* KO does not affect primary tumor growth in the 4T1 model; only metastasis is lost (Fig. 2). This suggests a change in cell migration out of the tumor, immune cell escape, and/or ability to colonize new tumors in other tissues (64). Angiogenesis was present in all tumors and categorized as mild in our analysis; others have reported 4T1 tumors display high levels of angiogenesis (65). However, since angiogenesis did not differ among tumor types, a change in angiogenesis is unlikely to be the cause of decreased metastasis in the KO tumors. Others have shown that NO can drive cell migration in tissue culture (66, 67) and a change in *in vivo* cell migration due to *NOS2* KO might provide an explanation for the changes in metastasis we find in the present study.

Here, immunoprofiling of primary tumors (Table 1) revealed high levels of MDSCs, tumor-associated macrophages (TAMs), but low levels of cytotoxic T-cells, consistent with an immunosuppressed tumor microenvironment for both tumors formed by WT and *NOS2* KO cells, and with large tumor growth. No major differences in the immune cell profile were detected in these tumors; all expressed IFNγ, a potent inducer of NOS2 in 4T1 cells as noted above. Immunosuppression in these tumors is therefore not due to tumor cell NOS2. Immunosuppression may, however, be due to PGE_2_ from tumor cells, which is known to be immunosuppressive (12, 13) and is constitutively high in both parental and *NOS2* KO 4T1 tumor cells (Fig. 3*F*). Immunosuppression may also be due to the high levels of TAMs and MDSCs in these tumors (68, 69), which can be recruited to the tumor by PGE_2_ or downstream effector molecules (70). Additionally, NO production increases PGE_2_ production by ∼50% (Fig. 3*F*), potentially further promoting immunosuppression and possibly affecting tumor cell escape into the blood stream or lymphatic system.

A previous study showed that inhibiting COX2 with indomethacin, a non-steroidal anti-inflammatory drug (NSAID), reduced both the primary tumor burden and metastatic spread in a 4T1 model (12). In the same study, the authors generated a *NOS2*^*−/−*^ mouse, thereby preventing NO production by host immune cells, and observed a further reduction in both primary tumor growth and metastasis. Together, these findings demonstrate that COX2-dependent inflammation and NOS2-derived NO from immune cells collectively promote TNBC disease progression (12). Here, we show that *NOS2* knockout *in tumor cells* has an even greater effect on metastasis but little effect on primary tumor growth. Taken together, a strategy of inhibiting COX2 alongside inhibiting both tumor and immune cell NOS2 might prove an efficacious breast cancer treatment strategy.

Inhibiting COX2 in cancer is an active area of research. COX2 is a bifunctional heme-containing protein that converts arachidonic acid to prostaglandin H_2_ (PGH_2_), which is then converted to several other inflammatory prostaglandins, the most abundant of which is prostaglandin E_2_ (PGE_2_) (47). PGE_2_ signals through G-protein coupled receptors EP1-4 and drives many cancers including breast cancer, leading to the use of aspirin and other NSAIDs in clinical trial both for preventing and for overcoming cancer. Additional trials are underway for drugs targeting receptors EP2 and EP4, the most prominent receptors in the tumor microenvironment (13). Importantly, PGE_2_ is immunosuppressive, inhibiting both T cell recruitment and enhancing MDSC infiltration (3, 14, 71, 72) and, additionally, can impair oxidative phosphorylation and glycolysis in immune cells (13).

To further understand how NOS2 alters tumor cells, we examined NO-dependent changes in metabolism and gene expression for 4T1 cells and a second cell line, human colorectal cell line DLD1. NO production leads to inhibition of oxidative phosphorylation and a glycolytic phenotype in both 4T1 and DLD1 cells (Fig. 3), another hallmark of aggressive tumors. Inhibition is likely occurring in at least two ways, through inhibition of cytochrome oxidase (complex IV) by tight binding of NO to heme (42, 43), and by inhibiting aconitase in the TCA cycle through tight binding of NO to the active site non-heme iron (28, 41), leading to loss of α-ketoglutarate (Fig. 3*E*). Metabolic reprograming to rely on glycolysis for ATP production leads to increased metabolites for DNA replication, energy production despite lower oxygen levels in the tumor interior, enhanced epithelial to mesenchymal transition (EMT), which leads to increased stem cell-like characteristics that aid in metastasis, epigenetic changes, and promotion of cell migration (7, 9).

Gene expression and genetic changes are likely correlated with tumor aggression as well. We and others have previously shown that NO inhibits both DNA and RNA demethylation, leading to an altered epigenetic landscape, another factor associated with aggressive cancers. Here, we show that endogenous NO in both 4T1 and DLD1 cells leads to an increase in m^6^A, increasing by ∼2.5-fold (Fig. 4*A*). Changes in DNA methylation and histone methylation are expected as well in response to endogenous NO.

Methylation alters DNA availability and RNA splicing, trafficking, translation, and degradation. Transfer RNA (tRNA) is also methylated and tRNA methylation is emerging as an important component of translational regulation (73, 74). Demethylation reverses these effects and is required for proper regulation of gene transcription and translation. The aberrant epigenetic landscape of tumors is now widely implicated in driving metastasis, although mechanistic insight is lacking and a possible role for regulation by NO unclear.

There are >100 demethylases in humans. The Fe(II)/2-oxoglutarate (OG)-dependent dioxygenase (2-ODD) family of DNA and RNA demethylases catalyze the removal of epigenetic methyl groups on DNA and RNA bases. All 2-ODD demethylases have a non-heme iron at their catalytic site and use 2-oxoglutarate (2OG, also called α-ketoglutarate) and O_2_ as co-substrates. We and others have demonstrated that NO inhibits the 2-ODD demethylases by binding to the non-heme iron in the enzyme’s catalytic site, forming a surprising dinitrosyl complex rather than the expected mononitrosyl complex with NO replacing O_2_ (5, 6). For RNA, this inhibition results in increased N^6^-methyladenosine (m^6^A) on mRNA of NO-regulated genes.

Our transcriptomics indicate >1200 genes are differentially expressed on comparing *NOS2*^*−/−*^ 4T1 cells to parental 4T1 cells in the presence of cytokine, but which—if any—of these are linked to 4T1 metastasis remains unclear. Our gene set enrichment analysis (GSEA) indicates that NOS2 is associated with DNA damage repair and cell cycle progression, pathways associated with aggressive tumors. Interestingly, genes associated with these pathways are down regulated in the presence of NO. Using two-color flow cytometry, we show that cytokine induces DNA damage as indicated through increased levels of γH2AX (Fig. 4), presumably through NO production. The DNA damage response (DDR) is designed to halt DNA replication through cell cycle checkpoint proteins until the damage is repaired and replication is safe to proceed (75). In cancer, DDR cell cycle checkpoints are often lost, allowing cancer cells to continue growing unabated while gaining genomic instability and fueling tumor progression (75, 76, 77), leading to considerable effort to develop drugs that exploit this loss in DDR (76). Our data now suggest there may be a direct NOS2-dependent link between genetic dysregulation and metastasis. How NO might lead to depletion of cell cycle checkpoint expression is not yet clear, but a potential outcome – that of genomic instability and continued cancer proliferation – would be a key driver of metastasis. One possible mechanism would be for the cell cycle checkpoint genes to be particularly sensitive to loss of RNA and DNA demethylation proteins through inhibition by NO.

In summary, we have demonstrated that *NOS2* knockout in a TNBC mouse model leads to loss of metastasis, the cause of 90% of cancer deaths (2). While the mechanism linking NO production to loss of lung metastasis remains unresolved, we find multiple and likely linked NOS2-dependent outcomes in TNBC that, taken together, may contribute, including inducing a glycolytic state, prostaglandin-dependent immunosuppression, and translational and genetic dysregulation. NO-dependent enhanced cell migration and/or angiogenesis, as described by others, may additionally contribute to poor outcomes.

## Experimental procedures

### Cell culture

Cell culture was undertaken as previously described (27). 4T1 cells were obtained from the University of Arizona Cancer Center Experimental Mouse Shared Resource (EMSR) facility, and DLD1 cells were purchased from the American Type Culture Collection (ATCC) and validated by those facilities using short tandem repeat analysis. Recombinant murine cytokines IFNγ (Cat. # 315-05) and IL1β (Cat. # 211-11B) were purchased from PeproTech. Recombinant human cytokines IFNγ (Cat. #80385), IL1β (Cat. #54059) and TNF⍺ (Cat. #16769) were purchased from Cell Signaling. Working stocks of murine and human cytokines were resuspended following manufacturer’s recommendations to 20 μg/ml and 100 μg/ml, respectively.

4T1 and DLD1 cells were cultured in Roswell Park Memorial Institute medium (RPMI 1640; Gibco) supplemented with 10% fetal bovine serum (FBS) and 1% penicillin-streptomycin (P/S; Gibco). Both cell lines were cultured at 37 °C in 5% CO_2_ with saturating humidity. In general, 300 k to 500 k cells were seeded into a six-well plate, grown to ∼80% confluency overnight, and treated the next day. Cells were washed twice with 1X calcium and magnesium free PBS (CMF-PBS) and treated with or without murine (IFNγ and IL1β), or human (IFNγ, IL1β, and TNFα) cytokines for 18 to 24 h in the appropriate serum-free medium supplemented with 1% P/S. For all experiments, murine cytokines were added at 20 ng/ml and human cytokines at 100 ng/ml, as previously described (27). When used, NOS2 inhibitor 1400W (Caymen Chemicals) was added from a DMSO stock solution (100 mM) to final concentration 20 μM. Aliquoted 1400W stock solutions were stored at −20 °C and used only once (refreezing led to loss of compound activity). Deviations in cell culture for specific measurements are described in each protocol.

### CRISPR/Cas9 NOS2 knock out

Homozygous knockout 4T1 cell lines for *NOS2* were created using CRISPR/Cas9 technologies in the University of Arizona Cancer Center (UACC) Genome Editing Facility. Double strand breaks were produced on either side of exon 4 of the *NOS2* gene at sites on mouse chromosome 11 with CRISPR guide RNAs (crRNAs) corresponding to the sequences 5′-CTTGGTTGACTCCACGGAGA-3′ and 5′-GTGTAACCGGTTTTGGATTC-3′. Parental 4T1 breast cancer cells were transfected with Cas9 protein, crRNAs, and trans-activating crRNA (Integrated DNA Technologies) using the Lipofectamine RNAiMAX reagent (Thermo Fisher Scientific). Two days after transfection, single cells were plated in five 96-well plates by the UACC Flow Cytometry Shared Resource. Colonies were expanded and, after approximately 3 weeks, clones were screened by PCR with primers internal to the deletion (5′-GTCTTGCTTGGGGTCCATCA-3′and 5′-CCCCGACTGGACAAAGGATG-3′) and primers spanning the deletion breakpoints (5′-CAGAGAGACGCCACAGTTGA-3′ and 5′-GAACGACCCCACGATGTAGA-3′). Clones that were positive for ligation-junction fragment and negative for sequences inside the targeted deletion were scored as potentially homozygous for the exon 4 deletion on agarose gels. Absence of NOS2 protein was confirmed by Western blot analysis. Two clones, A40 (KO1) and E3 (KO2), were selected for further analyses.

### Western blotting

For immunoblotting, cells were lysed in RIPA buffer (78) supplemented with 1% Protease Inhibitor Cocktail and Phosphatase Inhibitor Cocktail 3 (Sigma-Aldrich). Protein concentrations were determined using the BREAST CANCER assay procedure (Pierce), with bovine serum albumin as a standard. For cell-based measurements, 30 μg of total cellular protein was loaded per lane and separated by SDS/PAGE. After transfer, the nitrocellulose (Amersham) was blocked with 5% nonfat milk in 1X tris-glycine buffered saline with 0.1% Tween-20 (TBST). For the detection of phosphorylated proteins, membranes were blocked in 5% bovine serum albumin (BSA) in 1X TBST prior to addition of primary antibodies and followed with peroxidase-conjugated secondary antibodies. Validated commercial antibodies were used, listed in Table S1 and used as recommended by the vendor. Working concentrations are listed in the table. Images of the blots were captured using a SYNGENE G:Box gel imager. Full blots are shown in Figure S3.

### Real-time quantitative PCR

For RT-qPCR, RNA was isolated from cells using QIAzol Lysis Reagent (Qiagen) and RNeasy Mini Spin Columns (Qiagen), following manufacturers recommendation. cDNA was synthesized using 1 μg of RNA with Superscript II Reverse Transcriptase (Thermo Fisher) and random hexamer primers (Thermo Fisher) and assessed by RT-qPCR using SYBR Green Mastermix on an Applied Biosystems 7000 Real-Time PCR System (ABI). RT-qPCR conditions were: One cycle for 10 min at 94 °C; 40 cycles of 15 s at 94 °C, 30 s at 55 °C, and 1 min at 70 °C; and one cycle for 1 min at 70 °C, and 1 min at 60 °C. Results were analyzed using the 2^−ΔCt^ method and normalized to *GAPDH* or *β-actin*. Primers used are listed in *SI Appendix*, Table S2.

### Nitric oxide detection in live cells

Nitric oxide from live cells was measured in real time using the oxyhemoglobin detection method as previously described (27). Briefly, oxyhemoglobin was prepared by reducing bovine hemoglobin (Sigma-Aldrich) with dithionite (J. T. Baker) for 20 min, concentrated with an Amicon Ultra-15 Filter Unit (50 kDa cutoff) to remove dithionite, diluted in Tris buffer (pH 7.5), concentrated again, and flash frozen for later use. Cells were plated with a density between 0.1 × 10^6^ to 0.3 × 10^6^ cells per well in a 24-well plate and grown to ∼80% confluency overnight. For NO measurement, 4 μM of oxyhemoglobin in 1 ml of CMF-PBS supplemented with 1 mM of L-arginine was layered onto cells with each cell parameter examined in duplicate. Cells were maintained at 37 °C in a BioTek Synergy H1 plate reader and full UV-visible absorption spectral scans were obtained every 10 min for 60 to 90 min. At the completion of the experiment, DEA NONOate (20 μM, Cayman Chemicals) was added to release excess NO and oxidize the remaining oxyhemoglobin, followed by recording a final spectrum. Spectra were baseline corrected by subtracting the absorbance value at the longest wavelength (generally 650 nm) from all other values. To quantify NO reaction with oxyhemoglobin over time, the absorbance values at 401 nm and 421 nm, the wavelengths reflecting the maximal change between oxyhemoglobin and methemoglobin, were subtracted for each time point (ΔA_401-421_), normalized by subtracting the initial value from subsequent values (to account for spectral anomalies), and divided by the maximal value for ΔA_401-421_ (from DEA NONOate addition). Oxyhemoglobin autooxidation was estimated at each time point from wells lacking cytokine, which did not express NOS2, and subtracted out. Total cell number was estimated by total protein in the well (Micro BCA Protein Assay, Thermo Fisher) and divided through. The resulting data values were fitted to a straight-line equation, yielding pmol NO detected/min/mg total cellular protein.

### m^6^A measurement

For m^6^A measurements, total RNA was isolated from 10 million cells using QIAzol Lysis Reagent (Qiagen) following manufacturers recommendation. The RNA pellet was resuspended in lysis/binding buffer (from kit) to isolate Poly(A)+ RNA using the Magnetic mRNA Isolation Kit (New England Biolabs) following the manufacturers’ recommendation and quantified using a nanodrop device. 200 ng of mRNA was examined for m^6^A content using the ELISA-like colorimetric EpiQuick m^6^A RNA Methylation Quantification Kit (EpigenTek) and following the manufacturers recommendations.

### Seahorse metabolic measurement

ATP consumption in 4T1 and DLD1 cells was measured using the XF Real-Time ATP Rate Assay kit (Agilent) and measured on a Seahorse XFe96 Metabolic Analyzer following the manufacturer’s recommendations. In this assay, the extracellular acidification rate (ECAR), which results from glycolysis and oxidative phosphorylation, and the oxygen consumption rate (OCR), from oxidative phosphorylation, are measured in the absence or presence of mitochondrial inhibitors. From this, ATP from oxidative phosphorylation, ATP from glycolysis, and total ATP are calculated. ∼20,000 cells/well were grown for 4T1, 4T1 KO, and DLD1 cells. Cytokine and NOS2 inhibitor 1400W were added 18 h before measurement. 1400W was also added to the measurement media. Cell number was determined at the end of the experiment by adding Hoechst dye (2 μM) and counting with a Cytation I cell counter. Data were analyzed with the Agilent Seahorse Analytics and GraphPad Prism software packages.

### α-Ketoglutarate measurement

α-Ketoglutarate levels were measured by mass spectrometry in the Arizona Cancer Center Analytical Chemistry Shared Resource. Cells were grown as described above, detached using trypsin, counted (1.5 million cells), pelleted, flash frozen and stored at −80 °C until measurements were made. α-Ketoglutarate was extracted from cell pellets by addition of 300 μl methanol:chloroform:water (7:2:1). 10 μl of ^13^C5-labeled α-ketoglutarate (Sigma-Aldrich) in solution was added as an internal standard. Samples were vortexed, sonicated, and centrifuged (15,800 RCF, 4 °C for 5 min). The resultant supernatant (200 μl) was transferred to a new tube and 600 μl of methanol added. Following vortex mixing and centrifugation, the supernatant was transferred to a new tube and evaporated to dryness in a vacuum concentrator. Once dry, the residue was resuspended in 100 μl mobile phase A and transferred to autosampler vials with limited volume inserts. An injection volume of 10 μl was used.

Analysis was performed on an Agilent Ultivo triple quadrupole mass spectrometer running in ESI negative mode coupled to an Agilent 1290 Infinity II UPLC system. Chromatographic separation was achieved using a Phenomenex Luna C18 column (3 μm bead size, 100 × 4.6 mm) and a gradient system of water and acetonitrile, both with formic acid at 0.1% (v/v). α-Ketoglutarate eluted at 10.2 min. A calibration curve was prepared from an α-ketoglutarate stock solution of 1 mg/ml serially diluted to a range of 70 – 0.4 μM. and the internal standard evaluated at 33 μM. The mass transitions for α-ketoglutarate and the internal standard were monitored, with one transition used for quantification and the other for confirmation that the correct compound was identified. Amounts of α-ketoglutarate extracted from samples was determined using the standard curve and normalized against the proportion of internal standard recovered. Masses and collision voltages used are in Table S3.

### Prostaglandin E_2_ measurement

4T1, 4T1-KO and DLD1 cells were grown in 12 well plates overnight or until they achieved 80% confluency. Media was changed to serum free for 6 h followed by addition of cytokines and inhibitor or vehicle and incubated for overnight. Media was collected and PGE_2_ was measured immediately using the Prostaglandin E2 ELISA Kit (Cayman Chemical) and following the manufacturer’s recommendation. Data acquisition was with a BioTek Synergy H1 plate reader. Data were analyzed using the GraphPad Prism software package.

### PTEN activity measurement

PTEN activity was measured using competitive ELISA kit K-4700 (Echelon Biosciences Inc.) for detecting levels of phosphoinositol (4,5)-bisphosphate (PIP_2_) generated from added phosphoinositol (3,4,5)-triphosphate (PIP_3_). PTEN was isolated from cell lysate by immunoprecipitation (IP) and activity measured on bead following the manufacturer’s recommendations except for the following changes. Critical for our analyses was to remove dithiothreitol (DTT) from the IP and PIP_2_ generation steps so that any *S*-nitroso modification of the PTEN protein remained intact. Once PIP_2_ was generated, the recommended procedure (with DTT) was followed. Western blotting of IP material was conducted to ensure equal amounts of PTEN enzyme were isolated for each sample.

### Animal studies

Allograft studies were performed by the Arizona Cancer Center (AZCC) Experimental Mouse Shared Resource (EMSR) under the University of Arizona’s Institutional Animal Care Use Committee (IACUC) approval number 2021-0772. Female BALB/c mice were purchased from The Jackson Laboratory. 50,000 cells were injected into the fourth mammary fat pad of 8 mice for each cell line. Tumor burden was estimated using tumor volume according to the formula: [(width)_2_ x length]/2. All mice were euthanized around day 20 post 4T1 implantation by carbon dioxide (CO_2_) overdose, in accordance with IACUC-approved methods of providing rapid, painless, stress-free death. Mice were sacrificed on the same day when tumor burden including the primary, intraperitoneal, or lymph node became excessive (∼800–1000 mm^3^) or before if mice progressed to a moribund state.

### Pathology

A heart-lung pluck was collected from each mouse immediately after euthanasia. Lungs were inflated with neutral buffered formalin (NBF) and the pluck was immersed in NBF for at least 24 h. Following fixation, butterfly sections of lungs with heart and mediastinum were processed, embedded in paraffin, and 5-micron thick sagittal sections were stained with hematoxylin and eosin. Stained tissue sections were evaluated by an ACVP-certified veterinary pathologist. Lung metastases were enumerated and categorized by size as large (>500 microns in any one dimension), medium (150–500 microns), or small (<150 microns), and other tissue metastases of any size (mediastinal, paraoesophageal, cardiac, intravascular, or bronchial-associated lymphoid tissue) were enumerated as a separate category. Two independent experiments were conducted yielding a total of 13 to 17 mice per group. For the second set of experimental groups, two sections per lung were evaluated for each mouse and the total metastases from both sections were averaged. Primary tumor sections were stained and evaluated for angiogenesis and necrosis.

### Spectral flow cytometry

Single-cell suspensions were treated with a viability dye (ViaDye Red Fixable Viability Dye Kit, Cat #R7-60008, Cytek) for 25 min, washed to remove excess dye, and incubated with an Fc Block (Fc Shield, Cat. # 70-0161-U100, Tonbo/Cytek) for 10 min. After blocking, the cells were incubated with antibodies for extracellular markers (Cytek Custom Mouse Kit+). The cells were then prepared for intracellular staining using the Foxp3/Transcription Factor Staining Buffer Kit (Cat # TNB-0607-KIT, Tonbo/Cytek) and then incubated with antibodies for intracellular markers (Cytek Custom Mouse Kit+). After staining was completed, the cells were fixed, and all samples were run on the Cytek Aurora full spectrum flow cytometer equipped with five lasers (355, 405, 488, 561 and 640 nm excitation wavelengths), three scattering channels, and detection up to 64 fluorescence channels, housed in the Flow Cytometry and Immune Monitoring Shared Resource (FCIMSR) located in the University of Arizona Cancer Center. Data were analyzed using SpectroFlo (Cytek) and FlowJo software. All reagents used for the spectral flow cytometry analysis are listed in Table S4.

Guava DNA Damage Histone H2A.X Dual Detection Kit (Luminex) was used to measure DNA damage in 4T1 and DLD1 cells treated with cytokines and topoisomerase inhibitor etoposide (MilliporeSigma). 50,000 cells/well were grown in a six-well plate and media changed to serum and treated with vehicle, cytokines, or etoposide (100 μM) for 18 h. After trypsinization and pelleting, cells were collected in eppendorf tubes, fixed, permeabilized and stained with antibodies according to the manufacture’s recommendation. Flow analysis was carried out using a BD FACSCanto II.

### RNA-seq

RNA isolation, NGS library preparation, sequencing, and enrichment analysis were performed at the Arizona Genetics Core, Tucson AZ, RRID:SCR_012429. 1 to 1.5 million cells were grown under eight conditions in triplicate, pelleted, flash frozen, and stored at −80 °C. Total RNA was extracted from thawed cell pellets with the PureLink RNA Mini Kit (Invitrogen). RNA molecules were quantified on a Qubit 4 fluorometer using Qubit RNA Broad Range reagents (Invitrogen). RNA quality was assessed on a Bioanalyzer 2100 using Agilent RNA 6000 Nano reagents (Agilent). RNA libraries were prepared with a Watchmaker mRNA Library Prep Kit (Watchmaker Genomics) using 1000 ng total RNA input and indexed for multiplexing with xGen Stubby Adapter and UDI Primers for Element (IDT). Libraries were quantified on a Qubit 4 fluorometer using Qubit 1X dsDNA HS reagents (Agilent). Library size distributions were assessed on an AATI Fragment Analyzer using the HS NGS Fragment Kit (Agilent). The libraries were pooled to equimolar concentration, and a final round of QC was performed on the pool, as described for the individual libraries. The final pool was sequenced on an AVITI24 instrument using an AVITI 2x75 Cloudbreak Freestyle High Output Sequencing Kit (Element Biosciences).

Data were demultiplexed and adapter trimmed using Element's bases2fastq software version 2.2.0.1940753917. Reads were aligned to the GRCh38/GRCm38 reference genome using STAR v2.7.3a and read counts produced using STAR's GeneCounts function (79). Genes were annotated using the biomaRt package (80). Differential expression analysis was performed in R using edgeR exactTest (81). Gene Set Enrichment Analysis (GSEA) was performed using Gene Ontology biological process terms (55). Enriched gene sets were established with a false discovery rate adjusted *p*-value (q-value) <0.1.

### Statistical analysis

Plotting and statistical analyses were performed using GraphPad Prism version 10.6.1.

## Data availability

RNA-seq data are deposited with the gene expression omnibus (GEO) under entry GSE322659. All other data are included in the main text or supporting information file.

## Supporting information

This article contains supporting information.

## Conflict of interest

The authors declare that they have no conflicts of interest with the contents of this article.
