# Supplementary material for: Nitric oxide dependent metabolic and genetic dysregulation in metastatic triple-negative breast cancer
Source: J Biol Chem. 2026 Sep 8;302(10):113526. doi: 10.1016/j.jbc.2026.113526 (PMC13659492; doi:10.1016/j.jbc.2026.113526)
Supplement: Supplementary file 1 [file mmc1.pdf]

Supporting Information for

Nitric oxide dependent metabolic dysregulation, immunosuppression, and genomic instability in metastatic triple-negative breast cancer

Jennifer N Daw[c,d,1], Pricila K Moly[a,1], Nhi Van[a], Su Chung[a], Sarah Young[a], Sara Centuori[c], David G. Besselsen[e], Nathan Ellis[c,d,f], Ronald L. Heimark[b,c,d,2] and William R. Montfort[a,c,d,3]

[a]Department of Chemistry and Biochemistry, University of Arizona, Tucson, AZ, 85721; [b]Department of Surgery, University of Arizona, Tucson, AZ, 85721; [c]University of Arizona Cancer Center, Tucson, AZ, 85724; [d]CBIO Graduate Interdisciplinary Program, University of Arizona, Tucson, AZ, 85721; [e]University Animal Care, University of Arizona, Tucson, AZ, 85721; [f]Department of Cellular and Molecular Medicine, University of Arizona, Tucson, AZ, 85724.

[1]J.N.D. and P.K.M. contributed equally to this work
[2]Deceased
[3]To whom correspondence should be addressed. E-mail: montfort@arizona.edu.

This PDF file includes:

Tables S1-S7
Figures S1-S3

Table S1. Antibodies used for western blots

Antibody	Concentration	Species	Product No.
iNOS	1:2000	Rabbit	Cell Signal #13120
iNOS	1:500	Rabbit	Abcam #ab178945
Stat3	1:1000	Mouse	Cell Signal #124H6
P-Stat3 (Tyr705)	1:2000	Mouse	Cell Signal #4113
Stat1	1:1500	Rabbit	Cell Signal #9172
P-Stat1 (Ser727)	1:1500	Rabbit	Cell Signal #9177
NF-kB p65	1:1500	Mouse	Cell Signal #L8F6
P-NF-kB p65 (Ser536)	1:1500	Rabbit	Cell Signal #93H1
β-Actin	1:3000	Mouse	Cell Signal #3700
Monoclonal Anti-α-Tubulin	1:10000	Mouse	Cell Signal #3873S
Anti-rabbit IgG, HRP-Linked	1:2000	Goat	Cell Signal #7074
Anti-mouse IgG (H+L)-HRP Conjugate	1:2000	Goat	Bio-Rad #1706516
IRDye 800CW Donkey anti-rabbit IgG Secondary Anitbody	1:10000	Goat	Li-Cor #926-32213
IRDye 680RD Donkey anti-mouse IgG Secondary Anitbody	1:10000	Goat	Li-Cor #926-68072

Table S2. Primer sequences used for PCR

Primers	Forward (5’-3’)	Reverse (5’-3’)
mNOS2	TGTGACACACAGCGCTACAACA	GAAACTATGGAGCACAGCCACAT
mACTB	TCGCCCACACTGTGCCC	CCGGGATGGAGCCACCG

Table S3. Mass Spectrometry Masses and Fragmentation Parameters for α-Ketoglutarate

Compound name	Precursor (m/z)	Product (m/z)	Fragmentor (V)	Collision Energy (V)
^{13}C5 α-ketoglutaric acid	150.1	104.7	71	4
^{13}C5 α-ketoglutaric acid	150.1	60.1	71	8
α-ketoglutaric acid	145	101.1	70	4
α-ketoglutaric acid	145	57.1	70	8

Table S4. Reagents Used for Spectral Flow Cytometry

Specificity	Fluorophore	Company
CD45	V547	Cytek
CD4	V610	Cytek
CD3	R720	Cytek
CD25	BYG667	Cytek
CD8a	B515	Cytek
Ly6G	B548	Cytek
CD11b	R685	Cytek
F4/80	R780	Cytek
CD11C	V450	Cytek
iNOS + isotype control	BYG575	Cytek
TNFα + isotype control	BYG610	Cytek
IFNγ + isotype control	BYG710	Cytek
Foxp3	BV421	BioLegend #126419
Ly6C	BV785	BioLegend #128041
Granzyme B + isotype control	PerCP-Cy5.5	BioLegend #372211 / #400149
Arginase 1 +isotype control	PE-Cy7	ThermoFisher #25-3697-80 / #25-4321-82
Perforin + isotype control	APC	BioLegend #154403 / #400511
Viability Dye	ViaDye Red	Cytek
Fc Shield	NA	Cytek (Tonbo)
Foxp3 / Transcription Factor Staining Buffer Kit	NA	Cytek (Tonbo)

Table S5. Differentially Expressed Genes and Enriched Gene Sets[a]

Analysis	Enriched Gene Sets (+/-)[c]	Genes 3-Fold Enriched (+/-)	Total Genes Enriched
KO1 +cytokine vs 4T1 +cytokine[b]	14 / 0	594 / 661	1255
KO1 vs 4T1	0 / 0	240 / 704	944
KO1 vs KO1 +cytokine	18 / 131	230 / 560	790
4T1 +cytokine vs 4T1 +cytokine, +NOS2i	12 / 2	98 / 161	259
4T1 vs 4T1 +cytokine	70 / 107	534 / 609	1143
DLD1 +cytokine vs DLD1 +cytokine, +NOS2i	3 / 1	3 / 4	7
DLD1 vs DLD1 +cytokine	0 / 197	498 / 465	963

[a]FDR<0.10, P-value <0.005. [b]Cytokines used are IFNγ and IL1β (KO1 and 4T1) or IFNγ, IL1β, and TNFα (DLD1). NOS2i is inhibitor 1400W. [c]Gene sets enriched in the first cell line are listed as "+", in the second as "-".

Table S6. Enriched Gene Sets from RNA-seq Analysis (4T1 KO vs 4T1, +cyto)[a]

Cluster 1
GOBP_SPINDLE_CHECKPOINT_SIGNALING
GOBP_REGULATION_OF_MITOTIC_SISTER_CHROMATID_SEGREGATION
GOBP_NEGATIVE_REGULATION_OF_NUCLEAR_DIVISION
GOBP_NEGATIVE_REGULATION_OF_CHROMOSOME_ORGANIZATION
GOBP_MITOTIC_CELL_CYCLE_CHECKPOINT_SIGNALING
GOBP_DNA_INTEGRITY_CHECKPOINT_SIGNALING
GOBP_CHROMOSOME_SEPARATION
Cluster 2
GOBP_CELL_CYCLE_DNA_REPLICATION
GOBP_DNA_TEMPLATED_DNA_REPLICATION
GOBP_DNA_TEMPLATED_DNA_REPLICATION_MAINTENANCE_OF_FIDELITY
Cluster 3
GOBP_RNA_METHYLATION
GOBP_TRNA_METHYLATION

[a]From comparison of 4T1 NOS2 KO1 +cytokine vs 4T1 WT +cytokine

Table S7. Unique Enriched Genes from RNA-seq Analysis[a]

Cluster 1
ABRAXAS1 ACD ANAPC11 ANAPC15 ANAPC5 ANAPC7 APC ATF2 ATM ATR ATRIP ATRX AURKA AURKB BABAM1 BABAM2 BARD1 BAZ1B BECN1 BIRC5 BLM BMP7 BRCA1 BRCA2 BRCC3 BRD4 BRSK1 BUB1B BUB3 CCAR2 CCNB1 CCNB1-PS CCND1 CCNG1 CDC14B CDC16 CDC20 CDC23 CDC5L CDC6 CDCA8 CDK1 CDK5RAP2 CDK5RAP3 CDKN1A CDT1 CENATAC CENPE CEP192 CEP63 CHEK1 CHEK2 CHFR CHMP4C CLOCK CLSPN CRY1 CSNK2A1 CSNK2A2 CTC1 CUL3 CUL4A D7ERTD443E DCP2 DGKZ DIS3L2 DNA2 DONSON DOT1L DTL DTX3L DUSP1 DYNC1LI1 E2F1 EIF2AK4 EME1 EME2 ERCC1 ERCC4 ERCC6 ESPL1 ESR1 ETAA1 EXOSC10 FANCD2 FBXO31 FBXO4 FBXO43 FBXO5 FEM1B FGFR2 FGFR3 FOXN3 FOXO4 FZR1 GEN1 GIGYF2 GNB1L GNL3L H2AX H3F3A H3F3B HASPIN HINFP HNRNPU HSF1 HUS1 IER3 INCENP INIP INTS3 INTS7 KAT2B KAT5 KLHL22 KNL1 KNTC1 LCMT1 LIF LYN M1AP MAD1L1 MAD2L1 MAD2L1BP MAP3K20 MAPK14 MBD4 MBTPS1 MBTPS2 MCRS1 MDC1 MRE11A MRNIP MSH2 MTBP MUS81 NAA10 NABP1 NABP2 NAE1 NAT10 NBN NCAPD2 NCAPG2 NCAPH NCAPH2 NDC80 NEK1 NME6 NOP53 NOP53 NSMCE2 NUF2 NUMA1 ORC1 PABIR1 PARP1 PARP9 PIF1 PINX1 PLK1 PLK3 PLSCR1 PML POT1A POT1B PPP1R10 PPP2R1A PRKDC PRPF19 PRPF4B PSMG2 PTPN11 RAD1 RAD1 RAD17 RAD21 RAD50 RAD51 RAD9A RAD9B RB1 RBBP8 RECQL4 RECQL5 RFWD3 RHNO1 RINT1 RINT1 RNASEH2B RPA2 RPL24 RPS27L RPS6 RTEL1 SDE2 SETMAR SIRT1 SKA1 SKA3 SLX4 SMARCA5 SMARCAD1 SMC2 SMC4 SMG6 SPC24 SPC25 SPDL1 SRC STIL STK38 STN1 SYCP3 SYF2 TAOK1 TAOK2 TAOK3 TEN1 TERF1 TERF2 TERF2IP TEX14 TICRR TIMELESS TINF2 TIPIN TIPRL TNKS TOM1L1 TOM1L2 TOP2A TOP3A TOPBP1 TPR TRIM39 TRIP13 TRP53 TRP53BP1 TRRAP TTI1 UBE2C UFL1 UIMC1 USP28 USP44 VPS4A WAC WAPL WDR76 XPC XRCC1 XRCC3 XRCC4 ZFP207 ZFP207 ZFP207 ZFP207 ZFP207 ZFP830 ZFYVE19 ZW10 ZWILCH ZWINT
Cluster 2
AGER AICDA ASF1A ATAD5 ATG7 ATR ATRX BARD1 BLM BOD1L BRCA1 BRCA2 CCNE1 CCNE2 CDC45 CDC6 CDC7 CDK2 CDK9 CDT1 CENPS CENPX CHRAC1 CIZ1 DACH1 DBF4 DDX11 DNA2 DNAJA3 DONSON DYNLL1 E2F7 E2F8 EME1 EME2 ENDOG ETAA1 EXD2 EXO1 FAM111A FANCM FBH1 FBXO5 GEN1 GINS1 GINS2 GINS3 GINS4 GMNN HELB ILKAP INO80 MCM10 MCM2 MCM3 MCM4 MCM5 MCM6 MCM7 MCM9 MCMBP METTL4 MGME1 MMS22L MRE11A MTNAP1 MUS81 NBN NOC3L NUCKS1 NUGGC ORC1 ORC2 ORC3 ORC4 ORC5 ORC6 PARP1 PCNA POLA1 POLA2 POLD1 POLD2 POLD3 POLD4 POLE POLE2 POLE3 POLE4 POLG POLG2 POLN POLQ POLRMT PRIM2 PRIMPOL RAD50 RAD51 RBBP8 RECQL RECQL5 RFC1 RFC2 RFC3 RFC4 RFC5 RFWD3 RNASEH1 RPAIN RRM1 RRM2B RTEL1 RTF2 SAMHD1 SENP2 SETMAR SETMAR SMARCAL1 SSBP1 TICRR TIMELESS TIPIN TK1 TK2 TONSL TOPBP1 TRAIP TWNK UPF1 WDHD1 WDR18 WIZ WRN WRNIP1 ZFP830 ZMPSTE24 ZPR1 ZRANB3
Cluster 3
AKT1 ALKBH8 BCDIN3D BUD23 DALRD3 DIMT1 EMG1 FBL FDXACB1 FTSJ1 FTSJ3 HSD17B10 LARP7 LARP7-PS LCMT2 MEPCE METTL1 METTL14 METTL15 METTL16 METTL2 METTL3 METTL5 METTL6 METTL8 MRM1 MRM2 MRM3 MTO1 NOP2 NSUN2 NSUN3 NSUN3 NSUN4 NSUN5 NSUN6 RAMAC RBM15 RBM15B RNMT TARBP1 TFB1M TFB2M TGS1 THADA THUMPD2 THUMPD3 TRDMT1 TRMO TRMT1 TRMT10A TRMT10B TRMT10C TRMT112 TRMT12 TRMT13 TRMT1L TRMT2B TRMT44 TRMT5 TRMT6 TRMT61A TRMT9B TYW3 WDR4 WDR6 ZCCHC4

[a]From comparison of 4T1 NOS2 KO1 +cytokine vs 4T1 WT +cytokine

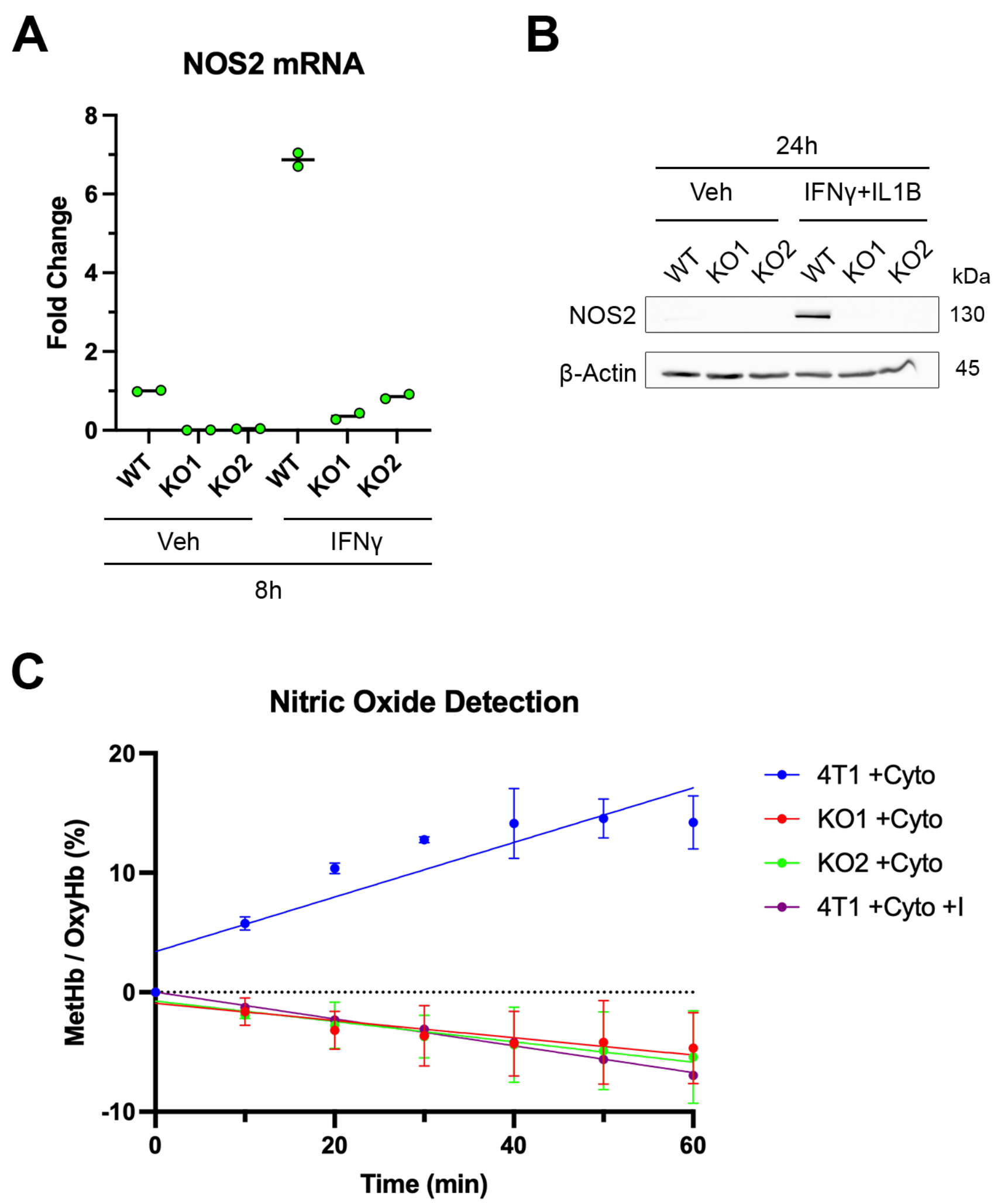

Fig. S1. Characterization of 4T1 *NOS2*$^{-/-}$ cell lines. (*A*) *NOS2* mRNA relative to β-actin measured by qPCR (2^ΔΔCT) indicate no *NOS2* message in KO1 and KO2. (*B*) Western blot indicates only wild type 4T1 cells express NOS2 in response to cytokine addition. (*C*) Real-time measurement of NO produced in tissue culture, monitoring NO conversion of oxyhemoglobin to methemoglobin spectroscopically. Only wild type 4T1 cells produce NO in response to cytokine addition. Inhibition of NOS2 by compound 1400W (+I) reverses NO production.

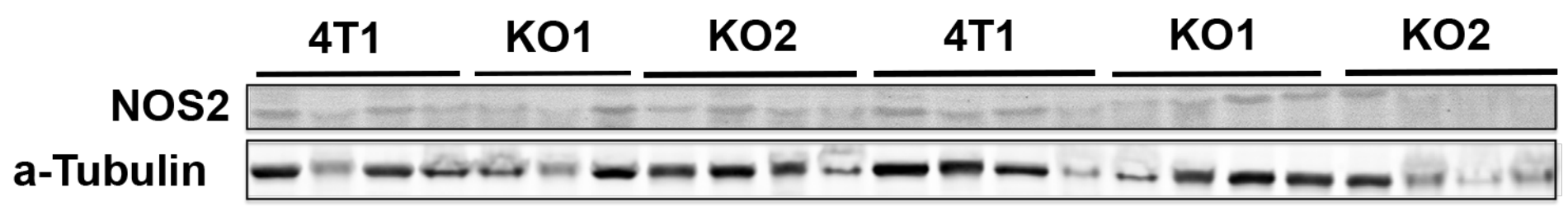

Fig. S2. Western blot of tumor lysates for NOS2. Tumor NOS2 is from tumor cells (4T1), immune cells, and other cells in the tumor microenvironment. Shown are blots for 3-4 tumors, in duplicate, and α-tubulin as loading control. The levels vary greatly, averaging 24.4 (8.2), 23.5 (4.5), and 29.0 (13.8) for the NOS2/tubulin ratio.

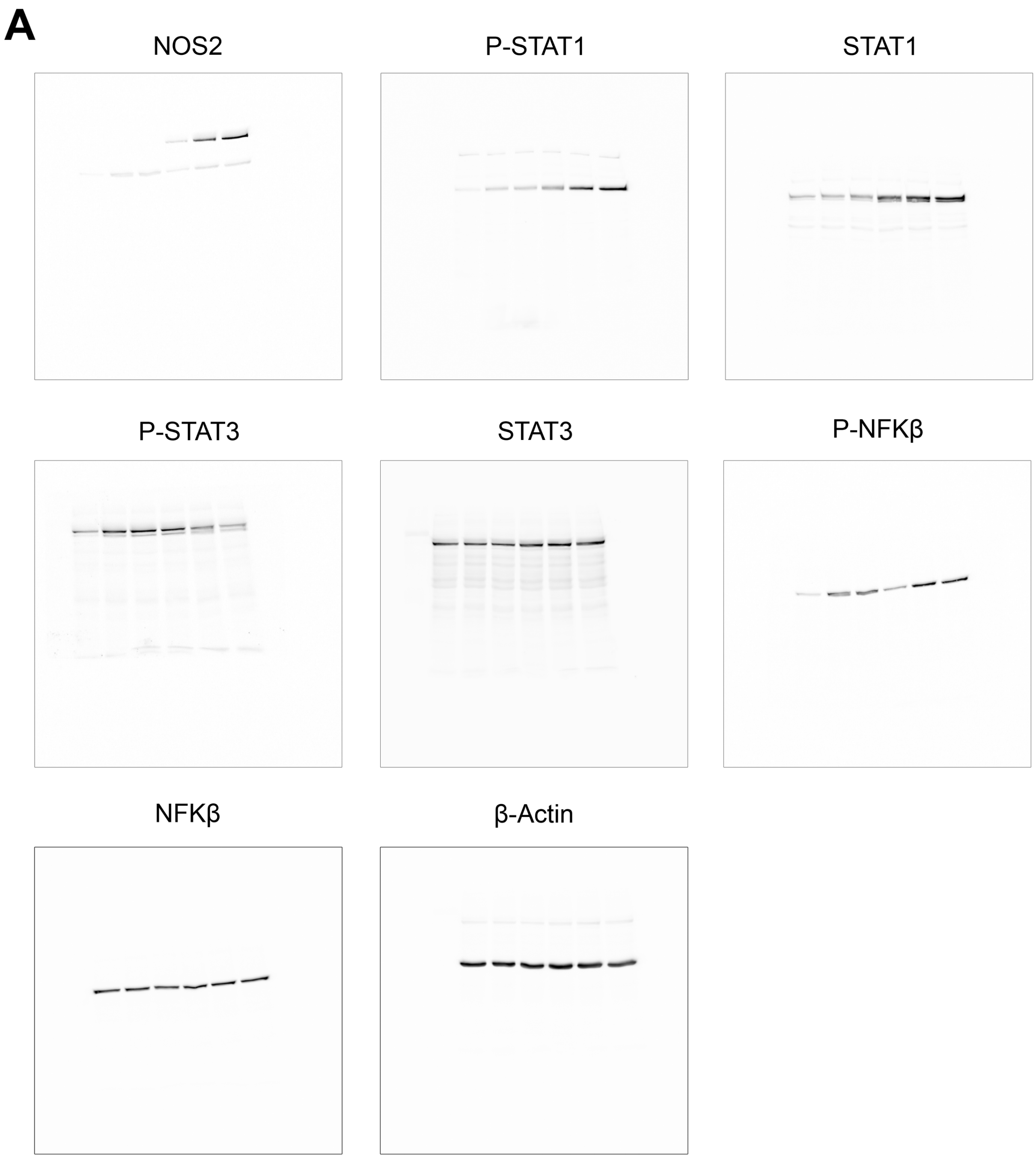
A
NOS2
P-STAT1
STAT1
P-STAT3
STAT3
P-NFKβ
NFKβ
β-Actin

B

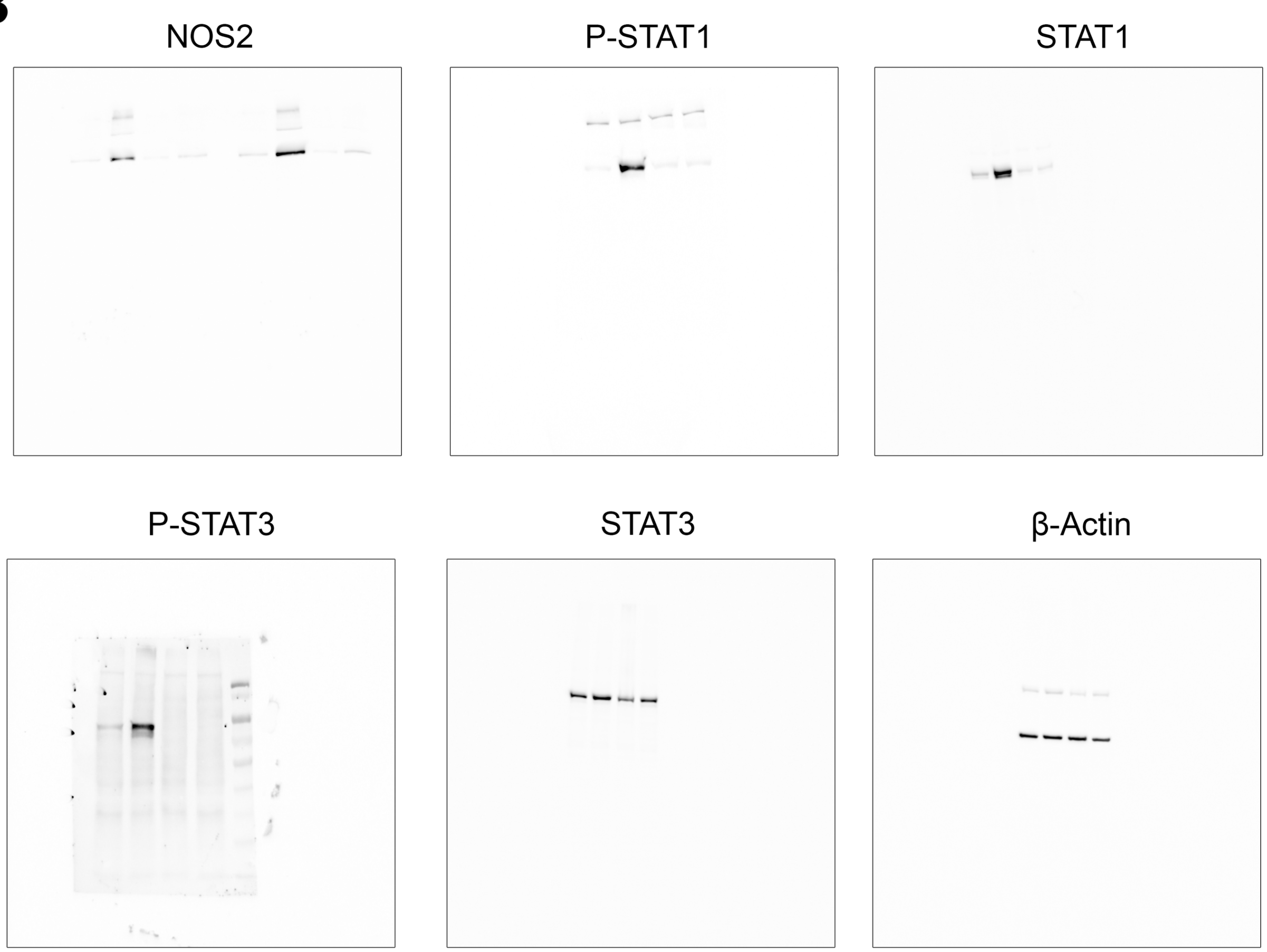

C

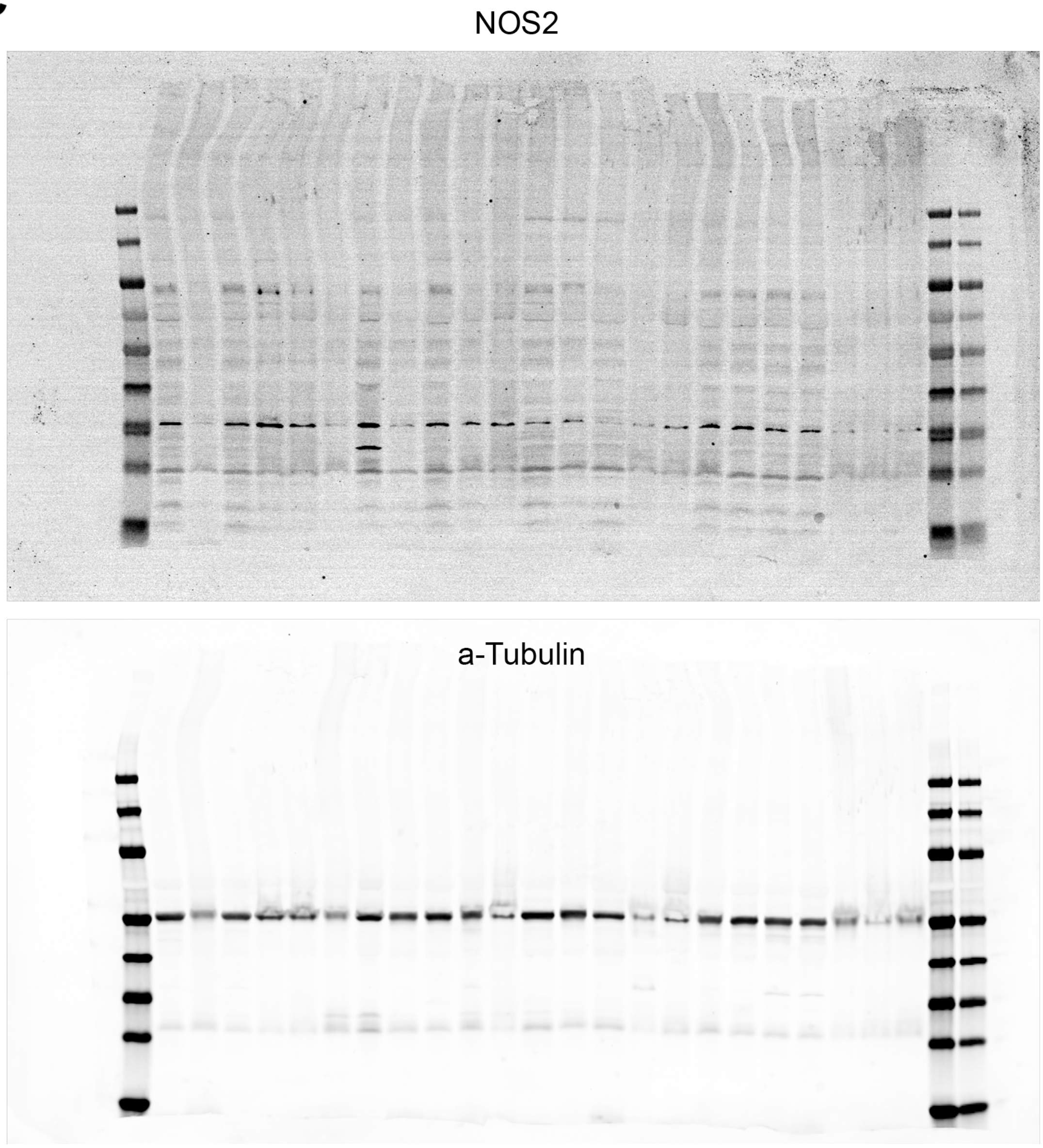

Fig. S3. Full western blots. (*A*) Fig. 1A. (*B*) Fig. 1C. (*C*) Fig. S2.
